# Whole genome sequencing, variant analysis, phylogenetics, and deep sequencing of Zika virus strains

**DOI:** 10.1038/s41598-018-34147-7

**Published:** 2018-10-26

**Authors:** Susmita Shrivastava, Vinita Puri, Kari A. Dilley, Erica Ngouajio, Jessica Shifflett, Lauren M. Oldfield, Nadia B. Fedorova, Lihui Hu, Torrey Williams, Alan Durbin, Paolo Amedeo, Sujatha Rashid, Reed S. Shabman, Brett E. Pickett

**Affiliations:** 1 J. Craig Venter Institute, Rockville, MD USA; 2 BEI Resources, ATCC, Manassas, VA USA; 3 J. Craig Venter Institute, La Jolla, CA USA; 4 Sharp Edge Labs, Pittsburgh, PA USA; 5 University of Maryland, College Park, MD USA; 6 American Type Culture Collection, Manassas, VA USA

## Abstract

The recent emergence of Zika virus (ZIKV) has been concentrated in the Caribbean, Southeastern United States, and South- and Central America; resulting in travel-based cases being reported around the globe. As multi-disciplinary collaborations are combatting the ZIKV outbreak, the need to validate the sequence of existing strains has become apparent. Here, we report high-quality sequence data for multiple ZIKV strains made publicly available through the National Institutes of Health- (NIH) funded biorepository, BEI Resources (www.beiresources.org). Next-generation sequencing, 3′ rapid amplification of cDNA ends (RACE), and viral genome annotation pipelines generated GenBank sequence records for 16 BEI Resources strains. Minor variants, consensus mutations, and consensus insertions/deletions were identified within the viral stocks using next-generation sequencing (NGS) and consensus changes were confirmed with Sanger sequencing. Bioinformatics analyses of the sequencing results confirm that the virus stocks available to the scientific research community through BEI Resources adequately represent the viral population diversity of ZIKV.

## Introduction

Zika virus (ZIKV) belongs to the *Flavivirus* genus within the *Flaviviridae* family, which also includes other arthropod-borne viruses including Dengue (DENV), West Nile (WNV), and Yellow Fever (YFV). ZIKV has a 10.7 kb positive-sense single-stranded genome, which codes for a single polyprotein that is co- and post-translationally cleaved into 10 mature proteins. ZIKV was initially isolated from a rhesus macaque in the Zika Forest of Uganda in 1947, and has subsequently been detected in sporadic human outbreaks in Africa, Southeast Asia, and the Pacific Islands prior to being identified in eastern Brazil in early 2015^1,2^. Since then, ZIKV has rapidly spread throughout the world with major foci across the western hemisphere^3,4^, including the southeastern United States^5^, as well as in Europe and southeast Asia^6^. Although transmitted primarily through the bite of infected *Aedes* mosquitos, ZIKV has also been detected in bodily fluids such as urine, breast milk, saliva, and semen. The capability of spreading through sexual transmission has also been established^7^. ZIKV infections are often asymptomatic or associated with relatively mild clinical signs and symptoms including fever, arthralgia, rash, and conjunctivitis. Guillain-Barre syndrome has been associated with ZIKV infection in adults^8^. The link to severe neurodevelopmental defects, such as microcephaly in fetuses, was one of the driving factors for the World Health Organization declaring ZIKV a global public health emergency^9,10^.

An impressive worldwide response has been mounted to combat the public health concerns associated with ZIKV infection. Prominent researchers in epidemiology, immunology, and other fields are forging together to better support the multitude of clinicians and patients that are battling the pathogen. In such an environment, communication and collaboration between investigators and institutions are imperative to success. In an effort to provide a central repository to store and distribute resources to researchers and specifically meet the emerging needs for research materials, the United States National Institute of Allergy and Infectious Diseases (NIAID) has established the Microbiology and Infectious Diseases Biological Resources Repository (MID**-**BRR), known publicly as BEI Resources (www.beiresources.org).

The purpose of this work is to authenticate the coding region sequence of ZIKV strains commonly used by the scientific community. We have generated consensus sequence and identified minor variants through deep sequencing on the ZIKV strains stored at BEI Resources. The methods and sequences reported here should minimize the cost and effort associated with independently validating these strains by individual laboratories as well as increase the pace of Zika-related discoveries.

## Methods

### RNA Extraction and Material Validation

ZIKV RNA was isolated using the RNeasy Mini kit (Qiagen) and eluted in 50 μl following a previously-described protocol^11^, and did not consist of additional virus passages prior to RNA isolation. ZIKV cDNA was synthesized from 5 μl undiluted RNA using SuperScript III First-Strand Synthesis kit (Thermo Fisher Scientific) followed by RNase H digestion. Quantitative real-time Polymerase Chain Reaction (qRT-PCR) was performed to confirm the presence of ZIKV genetic material using Taqman Universal 2X Master Mix II with UNG (Thermo Fisher Scientific), custom primers (IDT), and probes (Thermo Fisher Scientific). The qRT-PCR assay was performed with a Roche LightCycler 480 II by incubating the reactions at 50 °C for 2 min prior to incubation at 95 °C for 10 min followed by 50 cycles of denaturing at 95 °C for 15 seconds and elongation/extension at 60 °C for 1 minute. Data was collected at the 60 °C step.

### Genome Sequencing

Three sets of custom PCR primers were designed to generate amplicons from consensus Zika genome sequences and primers were diluted to 2 μM and pooled in equal volumes. Amplicon 1 was generated with primers 5′- GCTAACAACAGTATCAACAG-3′ and 5′- GATCTTTGTGGTCATTCTCTTC-3′. Amplicon 2 was generated with primers 5′- GTATGGAATGGAGATAAGGCC-3′ and 5′- ATGGTCTCTARGGTCTCCGG-3′. Amplicon 3 was generated with primers 5′- GTWGCATCTGCCGGAATAAC-3′ and 5′- GGCTGCACAGCTTTCCCCAA-3′. Three independent PCR reactions were performed on 2 μL of the reverse transcription products using Phusion 2x Hot Start Mix (New England Biolabs) to generate three overlapping ~3 kb amplicons across the genome. Amplicons were verified on 1% agarose gels. Amplicons were quantitated using a SYBR Green dsDNA detection assay (SYBR Green I Nucleic Acid Gel Stain, Thermo Fisher Scientific), and all three amplicons per genome were pooled in equal concentrations. The RNA-based and DNA-based sequence-independent single-primer amplification (SISPA) methods were used to generate complete genome sequence data for these BEI Resources strains with 300 bp paired-end reads on the Illumina MiSeq instrument.

For DNA SISPA, 50 to 200 ng of pooled amplicon DNA were combined with dimethyl sulfoxide and a random hexamer oligonucleotide with unique barcodes for each sample. The mixture was incubated at 95 °C for 5 min and immediately placed on ice. The denatured DNA template was then incubated with the Klenow fragment 3′−5′-exo (New England BioLabs) at 37 °C for 60 min, followed by 75 °C for 10 min. The resulting DNA was amplified by PCR using AmpliTaq Gold (Life Technologies) for 35 cycles (94 °C for 30 s, 55 °C for 30 s, and 68 °C for 45 s). PCR mixture contained primers specific for each barcode with either 3 or 4 N’s at the 5′ end using an equimolar ratio combination. The resulting DNA was then treated with exonuclease I (New England BioLabs) at 37 °C for 60 min.

For RNA SISPA, 5 μl of viral RNA and uniquely barcoded random hexamer oligonucleotides were combined with SuperScript III reverse transcriptase/Platinum Taq high-fidelity enzyme mix (ThermoFisher Scientific) for first-strand cDNA synthesis (50 °C, 5 minutes 0.4 °C, 5 minutes, 25 °C, 15 minutes, 50 °C, 30 minutes, 55 °C, 10 minutes, 70 °C, 15 minutes, 4 °C, 10 minutes-1 h). The cDNA (first strand) was incubated with RNase H and Klenow fragment 3′−5-exo (New England Biolabs) at 37 °C for 60 min, followed by 80 °C for 10 min for second-strand DNA synthesis. The product was then amplified by polymerase chain reaction for 45 cycles (94 °C for 30 s, 55 °C for 30 s, and 68 °C for 30 s). The PCR mixture contained primers specific for each barcode with either 3 or 4 N’s at the 5′ end using an equimolar ratio combination.

The DNA or RNA SISPA products were then normalized and pooled into a single reaction mixture that was purified using a QIAquick PCR purification kit (Qiagen). SPRIselect (Beckman Coulter) bead selection method was used to select for SISPA products that were 300 to 1000 bp in length prior to sequencing.

### 3′ Race

3′ Rapid Amplification of cDNA Ends (RACE) was performed by poly-adenylating the ZIKV RNA using E-PAP (Thermo Fisher Scientific) followed by cDNA synthesis and PCR using FirstChoice RLM-RACE Kit (Thermo Fisher Scientific). The sequence of the ZIKV-specific primer used at the PCR step was 5′-AGAGTGTGGATTGAGGAGAACGAC-3′. PCR products were then cloned and sequenced with Sanger technology prior to incorporation into the respective consensus genome sequence. Sequences obtained by 3′ RACE extended those obtained by NGS by approximately 70 nucleotides.

### Read Assembly

After sequencing, reads from each sample were deconvoluted by barcode and trimmed to eliminate low-quality regions consisting of a Phred score <30 with an ascii offset of 33, minimum length of 64, and minimum nucleotide quality score of 20. SISPA hexamer primers and barcode sequences were also removed in the trimming process. Trimmed reads were subjected to *de novo* assembly with CLC Bio software. The resulting contigs were then queried against a custom full-length Zika virus reference database to determine the closest reference sequence. Contigs were then mapped to the selected reference sequence for each sample using the CLC Bio software suite. Parameters for this reference-based assembly consisted of mismatch cost, 2; gap cost, 3; reads mapping to multiple loci, placed randomly; fraction of the read that must be placed for assembly, 0.5; similarity in the fraction of the read to be placed, 0.95; paired read information, not used; alignment mode, local. For sites where the majority of reads disagreed with the sequence from the reference strain, the reference sequence was updated accordingly to improve read mapping in subsequent assemblies. A final mapping of all next-generation reads to the selected reference sequences was then performed within the CLC Bio software. Curated assemblies were validated and annotated with the Viral Genome ORF Reader (VIGOR) version 3 annotation software^12^ before submission to GenBank. VIGOR was used to predict genes, perform alignments, ensure the fidelity of open reading frames, correlate nucleotide polymorphisms with amino acid changes, and detect any potential sequencing errors. The annotation was subjected to manual inspection and quality control before submission to GenBank.

### Consensus Variation

The nucleotide and translated protein sequences obtained from various virus stocks provided by BEI Resources were grouped together with GenBank sequences having the same strain name. Each group of sequences was then aligned using MAFFT^13^, and checked for any observed differences at the consensus level across all accession numbers for the same strain. Each assembled position that differed from the previously-published sequences were manually inspected and verified.

### Sanger sequencing

Viral RNA was purified from isolates as described above. cDNA was generated using the reverse transcriptase Superscript III Supermix (ThermoFisher Scientific) and random hexamers according to the manufacturer’s standard protocol. Two viral amplicons were amplified using two sets of ZIKV-specific primers: (i) primer 4F–5′-GCACAGGAYAARCCRACTGT-3′ and primer 7R–5′-TTCATCTCGATYAGRTGRGC-3′ (2072 bp; positions 1080–3151 using PRVABC59 numbering) and ii) primer 21F–5′-GAAGTGGAAGAARCACGGAC-3′ and 21R–5′-TTCAAATATTGCCCCYARTG-3′ (783 bp; positions 8130–8912 using PRVABC59 numbering). All of the PCR products were amplified using 3 µl of 10 µM primers, 15 µl of 2X Q5 Hot Start High-Fidelity Master Mix (New England BioLabs), 8 µl of nuclease-free water and 4 µl of cDNA with the following thermocycler conditions: initial denaturation at 98 °C for 30 seconds; 98 °C for 5 seconds, 50 °C for 20 seconds and 72 °C for 2 minutes and 30 seconds (5 cycles); 98 °C for 5 seconds, 55 °C for 20 seconds and 72 °C for 2 minutes and 30 seconds (35 cycles); and a final extension step at 72 °C for 5 minutes. The amplicons generated using primers 4F and 7R were visualized on an agarose gel using ethidium bromide and a blue-light transilluminator, and target bands were purified with the QIAQuick Gel Extraction Kit (Qiagen). Purified PCR amplicons using both primer pairs were then sequenced using the Sanger method (GENEWIZ).

### Minor Variant Detection

Deep sequencing analysis was performed on all strains that were processed at the J. Craig Venter Institute (JCVI) to identify the minor variants circulating in the population. This involved generating a consensus sequence from all sequence reads for each sample using CLC mapping assembly (clc_ref_assemble_long) as discussed above. A custom script was then used to map all of the high-quality trimmed sequence reads from each viral strain to the associated consensus sequence by parsing the assembly output. Separate scripts were then used to calculate the major and minor alleles, determine the nucleotide location of the observed allele(s), and predict whether the change in each codon would result in an amino acid substitution. These annotations were then used to provide biological context and meaning to all detected minor variants. To find statistically significant variations in the population, all forward and reverse reads covering each position were checked and a statistical model using a binomial distribution was generated to ensure that each minor variant was observed above a specified frequency threshold (3%) with a 95% confidence interval followed by multiple-hypothesis correction using the Bonferroni method. Positions lacking sufficient coverage to call a minor allele with 95% confidence (p < 0.05) were not reported in the output. The variations observed in the sequencing reads for each strain were reported against the consensus sequence of the same strain to get the percentage of minor alleles in the population.

### Recombination Detection

The 16 new ZIKV sequences were combined with 90 publicly-available ZIKV sequences in GenBank, aligned with MAFFT^13^, and subjected to recombination analysis using RDP4^14^. The recombination detection methods included in the software suite were RDP^15^, GENECONV^16^, MaxChi^17^, CHIMAERA^18^, SiScan^19^, and 3SEQ^20^. Only recombination events with a Bonferroni-corrected p-value <= 0.01, which were confirmed by at least four methods were reported.

### Sequence Analysis

A correlation analysis between the root-to-tip genetic divergence and date of sampling was performed in Path-O-Gen v.1.4 with multiple sequence alignments generated with MAFFT^13^. The analyses were done separately for the Asian (n = 86) and the African (n = 20) lineages as well as for a combined dataset. Linear regressions of root-to-tip divergence as a function of sampling time were also performed with Path-O-Gen.

To perform the analysis, coding complete regions from 106 ZIKV genomes were extracted from the National Center for Biotechnology Information (NCBI) representing 86 and 20 sequences from the Asian and African lineages, respectively. The coding regions were aligned with MAFFT and manually trimmed^13^. Maximum likelihood (ML) phylogenies were reconstructed using the heuristic tree search algorithm implemented in PhyML^21^. ML bootstrapping was performed with 100 replicates to assess the robustness of tree topologies. A general time reversible (GTR) nucleotide substitution model with a proportion of invariant sites were used to generate the Maximum Likelihood tree.

Bayesian Evolutionary Analysis Sampling Trees (BEAST v.1.8.2) was used to calculate the phylogenetic relationships and time to the most recent common ancestor for the coding region of all 106 ZIKV sequences^13,22,23^. The necessary parameters and data were prepared in XML format using BEAUti version 1.8.3. Sequences were separated according to Asian or African lineage and the dates of strain isolation were pulled either from GenBank or from the peer-reviewed literature. The GTR substitution model was selected with all estimated base frequencies and Gamma + Invariant site heterogeneity model. BEAST was run using both strict and relaxed uncorrelated models. Multiple combinations of molecular clock and coalescent models were run for the chain length of 100 million with a tree sampling every 10000 chains. The time to the most recent common ancestor and the nucleotide substitutions per site per year was calculated. Using Tracer v.1.6^24^, the Markov Chain Monte Carlo (MCMC) steps and convergence of the runs were visualized and the Bayesian MCMC trees were generated using TreeAnnotator with 10% burn-in. The MCMC tree was visualized and edited in Figtree v.1.4.0 (http://tree.bio.ed.ac.uk/software/figtree/).

Reconstructing the changes in effective population size over time was performed with Bayesian skyline using the GMRF Bayesian Skyride model, followed by model evaluation using AICM values in the Tracer program^23^. The differences in the AICM values showed that the coalescent GMRF Skyride model was superior than the coalescent Bayesian Skyline Piecewise-constant population model^25^.

Selection pressure in the coding region was calculated on the same set of 106 ZIKV genomes, consisting of the 16 sequences from this study together with others in GenBank, using the SLAC, FEL, IFEL, and MEME algorithms within the HyPhy package^26^. Two sequences (NC_012532 and KF383118) were removed from the selection analysis due to multiple insertions/deletions (indels) causing frameshifts in the codon alignment. Nine identical sequences were subsequently removed prior to applying default parameters to the analysis including: global dN/dS value estimated at 1.0 and significance level of 0.05.

### Code Availability

With the exception of the commercially-licensed programs, all custom scripts used to identify minor variants are available in the Elvira project through SourceForge.

## Results

### Sequence Comparison of Various Stocks

To allow researchers to efficiently apply molecular biology methods on ZIKV genetic material, we used next-generation sequencing to generate coding-complete consensus genome sequences for 16 ZIKV strains available at BEI, without additional passaging. These strains represent historical samples belonging to the African lineage^27^, as well as strains within the Asian lineage from the ongoing ZIKV outbreak (Supplementary Table S1). These virus stocks were provided to JCVI by BEI Resources for sequence validation and authentication on behalf of the scientific research community. The ZIKV coding-complete sequence data from this report enhances our understanding of the epidemiological and evolutionary dynamics of ZIKV from the recent outbreak. We subsequently performed additional downstream bioinformatics analyses of these assembled sequence data to better understand how the reagents available from BEI Resources compare to earlier passages of similar material, and to determine how these sequences relate to all other ZIKV strains with publicly-accessible genomes.

The four African lineage samples that were sequenced in this study were provided by BEI Resources. These included the MR-766 strain from Uganda (ZIKV/Macaca mulatta/UGA/MR-766_SM150-V8/1947; BEI Resources: NR-50065; GenBank: KU963573), which is a separate stock from the previously-deposited prototype strain of Zika virus (GenBank: NC_012532.1); the IbH-30656/1968 strain (ZIKV/Homo sapiens/NGA/IbH-30656_SM21V1-V3/1968; BEI Resources: NR-50066; GenBank: KU963574); and two stocks of the DAK-AR-41524/1984 strain (ZIKV/Aedes africanus/SEN/DAK-AR-41524_A1C1-V5/1984; BEI Resources: NR-50338; GenBank: KY348860) and a separate passage (ZIKV/Aedes africanus/SEN/DAK-AR-41524_A1C1-V2/1984; BEI Resources: NR-50338; GenBank: KX198134). We compared the consensus sequences derived from different stocks of the same isolate to investigate the inherent variations produced by passaging of the same virus.

The MR-766 virus (BEI Resources: NR-50065) was isolated from the blood of a sentinel rhesus monkey in the Zika forest near Entebbe, Uganda, on April 20, 1947. Additional genomic sequences for MR-766 have been previously determined: KU720415.1, KX830960.1, KX377335.1, LC002520.1, DQ859059.1, KX601169.1, and AY632535/NC_012532. The GenBank accession number for the sequence of this isolate reported in this study, KU963573.2, complements these previous sequence data. The sequence for DQ859059.1 is very distinct from the rest of the MR-766 sequences, therefore it was not included in the comparison. Across all seven stocks from this isolate, we observed differences in 23 positions and six insertions/deletions (indels) at the nucleotide level (Table 1) as well as 17 substitutions and one indel at the amino acid level (Table 2). Fifteen of these substitutions were only observed in the reference sequence NC_012532.1 compared to all the other sequences from this isolate (Table 2). We found an insertion at position 118 followed by a deletion at position 139, resulting in a short frameshift within the reference sequence NC_012532.1 compared to all the other MR-766 isolates in NCBI, including the KU963573 sequenced at JCVI (Table 1). Similarly, a 12-base indel at genome position 1433 in NC_012532.1 was found for most sequences, including a deletion at this locus in the KU963573 sequence. The other four virus genomes KU720415.1, KX830960.1, KX377335.1 and LC002520.1, all reported a 12-base insertion at this position in the Envelope (E) protein. Similar comparisons were performed at the nucleotide (Supplementary Tables S2–S3) and amino acid (Supplementary Table S4) for other sequences in the African lineage. Coverage plots for the African-lineage sequences are also reported (Supplementary Figs S1–S4).

**Table 1:** Consensus substitutions in the genome of different UGA/MR-766/1947 sequences, with numbers representing the nucleotide position in the genome for each sequence.

Alignment position	NC_012532.1_NR-50065	KU720415.1	KX830960.1	KX377335.1	LC002520.1	JCVI_KU963573.2_NR-50065*	KX601169.1
118	1 base insertion of C at 118; after 117						
139	1 base deletion of T after 138						
721	A at 720	A at 713	A at 720	G at 720	A at 720	A at 712	A at 684
1100	C at 1099	T at 1092	T at 1099	T at 1099	T at 1099	C at 1091	T at 1063
1388	T at 1387	T at 1380	T at 1387	T at 1387	T at 1387	C at 1379	T at 1351
1432	T at 1431	T at 1424	T at 1431	T at 1431	C at 1431	T at 1423	T at 1395
1435.0.1446	12 base deletion after 1433	12 base insertion at 1427.0.1438 compared to NC_012532.1	12 base insertion at 1434.0.1445 compared to NC_012532.1	12 base insertion at 1434.0.1445 compared to NC_012532.1	12 base insertion at 1434.0.1445 compared to NC_012532.1 and T at 1443 in insertion instead of C	12 base deletion after 1425	12 base deletion after 1397 in consensus; also has reads with no deletion
1464	G at 1451	A at 1456	A at 1463	A at 1463	A at 1463	A at 1443	A at 1415
1562	C at 1549	C at 1554	C at 1561	T at 1562	C at 1561	C at 1541	C at 1513
1670	C at 1657	C at 1662	C at 1669	C at 1669	T at 1669	C at 1649	C at 1621
1825	G at 1812	A at 1817	A at 1824	A at 1824	A at 1824	A at 1804	A at 1776
3065	G at 3052	G at 3057	G at 3064	A at 3064	G at 3064	G at 3044	G at 3016
4472	C at 4459	T at 4464	T at 4471	T at 4471	T at 4471	T at 4451	T at 4423
4520	C at 4507	C at 4512	C at 4519	C at 4519	T at 4519	C at 4499	C at 4471
5178	C at 5165	C at 5170	C at 5177	T at 5177	C at 5177	C at 5157	C at 5129
5457	A at 5444	T at 5449	T at 5456	T at 5456	T at 5456	T at 5436	T at 5408
5513	C at 5500	C at 5505	C at 5512	C at 5512	T at 5512	C at 5492	C at 5464
6338	T at 6325	C at 6330	C at 6337	C at 6337	C at 6337	C at 6317	C at 6289
6353	T at 6340	G at 6345	G at 6352	G at 6352	G at 6352	G at 6332	G at 6304
7173	1 bp gap of T after 7159	T at 7165	T at 7172	T at 7172	T at 7172	T at 7152	T at 7124
7183	1 bp insertion of G after 7168	1 base gap after 7174	1 base gap after 7181	1 base gap after 7181	1 base gap after 7181	1 base gap after 7161	1 base gap after 7133
7852	A at 7838	C at 7843	C at 7850	C at 7850	C at 7850	C at 7830	C at 7802
7865	A at 7851	T at 7856	T at 7863	T at 7863	T at 7863	T at 7843	T at 7815
8303	G at 8289	C at 8294	C at 8301	C at 8301	C at 8301	C at 8281	C at 8253
8394	A at 8380	A at 8385	A at 8392	G at 8392	A at 8392	A at 8372	A at 8344
10140	C at 10126	C at 10131	C at 10138	C at 10138	C at 10138	T at 10118	C at 10090
10345	T at 10331	T at 10336	T at 10343	T at 10343	T at 10343	C at 10323	T at 10295
10696	A at 10682	C at 10687	A at 10694	A at 10694	A at 10694	A at 10674	A at 10646
		Missing 34 bases at 3′ end compared to NC_012532.1					Missing 110 bases at 3′ end compared to NC_012532.1

^*^Sequenced at JCVI as part of the current work.

**Table 2:** Substitutions in the polyprotein of different UGA/MR-766/1947 sequences, with numbers representing the amino acid positions in the polyprotein for each sequence.

Alignment position	NC_012532.1_NR-50065	KU720415.1	KX830960.1	KX377335.1	LC002520.1	JCVI_KU963573.2_NR-50065*	KX601169.1
6	E at 6	K at 6	K at 6	K at 6	K at 6	K at 6	K at 6
7	E at 7	K at 7	K at 7	K at 7	K at 7	K at 7	K at 7
8	I at 8	S at 8	S at 8	S at 8	S at 8	S at 8	S at 8
9	R at 9	G at 9	G at 9	G at 9	G at 9	G at 9	G at 9
10	R at 10	G at 10	G at 10	G at 10	G at 10	G at 10	G at 10
11	I at 11	F at 11	F at 11	F at 11	F at 11	F at 11	F at 11
205	H at 205	H at 205	H at 205	R at 205	H at 205	H at 205	H at 205
442…444		insertion after 441 of IVND	insertion after 441 of IVND	insertion after 441 of IVND	insertion after 441 of TVND		
446	I at 442	T at 446	T at 446	T at 446	I at 446	I at 442	I at 442
453	D at 449	N at 453	N at 453	N at 453	N at 453	N at 449	N at 449
573	R at 569	K at 573	K at 573	K at 573	K at 573	K at 569	K at 569
1784	N at 1780	Y at 1784	Y at 1784	Y at 1784	Y at 1784	Y at 1780	Y at 1780
2356	M at 2352	Y at 2356	Y at 2356	Y at 2356	Y at 2356	Y at 2352	Y at 2352
2357	H at 2353	A at 2357	A at 2357	A at 2357	A at 2357	A at 2353	A at 2353
2358	G at 2353	W at 2358	W at 2358	W at 2358	W at 2358	W at 2353	W at 2353
2582	I at 2578	L at 2582	L at 2582	L at 2582	L at 2582	L at 2578	L at 2578
2586	E at 2582	V at 2586	V at 2586	V at 2586	V at 2586	V at 2582	V at 2582
2732	C at 2728	S at 2732	S at 2732	S at 2732	S at 2732	S at 2728	S at 2728

^*^Sequenced at JCVI as part of the current work.

JCVI received 12 samples from BEI Resources that belong to the Asian lineage, which included the historical MYS/P6-740/1966 strain (ZIKV/Aedes aegypti/MYS/P6-740/1966; GenBank: KX694533), the more recent PRI/PRVABC59/2015 (ZIKV/Homo Sapiens/PR/PRVABC59/2015; BEI Resources: NR-50240; GenBank: KX087101), and other strains from the recent outbreak including: COL/FLR/2015 (ZIKV/Homo Sapiens/COL/FLR/2015; GenBank: KX087102), HND/R103451/2015 (Zika virus ZIKV/Homo sapiens/HND/R103451/2015; GenBank: KX694534), MEX/MEX_I-44/2016 (Zika virus ZIKV/Aedes aegypti/MEX/MEX_I-44/2016; GenBank: KY648934), MEX_2-81/2016 (ZIKV/Aedes.sp/MEX/MEX_2-81/2016; GenBank: KX446950), and MEX/MEX_I-7/2016 (ZIKV/Aedes.sp/MEX/MEX_I-7/2016; GenBank: KX446951).

The PRI/PRVABC59/2015 virus was originally isolated from the blood of a human in Puerto Rico during December 2015. The sequence generated from this stock in the current study (KX087101) was compared with the other existing sequences for PRVABC59 from NCBI (KU501215, KX601168, and KX377337). Only three substitutions and one indel were observed among all four whole genome sequences as shown in Table 3, which resulted in a total of two differences at the amino acid level, one each in the Envelope (E) and NS1 protein as shown in Table 4. The consensus sequence generated by JCVI is shorter by 28 and 1 bases for KX087101.3 at the 5′ and 3′ ends, respectively, compared to KX377337. These differences can primarily be attributed to SISPA-based methods not yielding sufficiently high coverage at the end of the template to generate a high-quality consensus sequence. Similar comparisons were performed at the nucleotide and amino acid levels (Supplementary Tables S5–S11) for other sequences in the Asian lineage. Coverage plots for the Asian-lineage sequences are also reported (Supplementary Figs S5–S16), as are the summary coverage statistics for all sequences in this study (Supplementary Table S12).

**Table 3:** Consensus substitutions in different PRI/PRVABC59/2015 sequences, with numbers representing the nucleotide position in the genome for each sequence.

Alignment position	KX377337.1	KU501215.1_NR-50240	JCVI_KX087101.3_NR-50240*	KX601168.1
1965	G at 1965	G at 1964	T at 1937	T at 1947
2781	G at 2781	T at 2780	T at 2753	T at 2763
8283	T at 8283	C at 8282	C at 8255	C at 8265
		Missing 1 base at 5′ and 131 base at 3′ end compared to KX377337.1	Missing 28 base at 5′ and 1 base at 3′ end compared to KX377337.1	Missing 18 base at 5′ and 110 base at 3′ end compared to KX377337.1

^*^Sequenced at JCVI as part of the current work.

**Table 4:** Substitutions in the polyprotein of different PRI/PRVABC59/2015 sequences, with numbers representing the amino acid positions in the polyprotein for each sequence.

Alignment position	KX377337.1	KU501215.1_NR-50240	JCVI_KX087101.3_NR-50240*	KX601168.1
620	V at 620	V at 620	L at 620	L at 620
892	G at 892	W at 892	W at 892	W at 892

^*^Sequenced at JCVI as part of the current work.

To computationally confirm the results of the consensus variations, we reviewed the assembled reads and did not observe any reads that extend into the region containing a deletion in the relevant strains. To further validate our results with laboratory methods, we performed Sanger sequencing on two amplicons (spanning positions 1080–3151 and 8130–8912) that include representative indels and nucleotide substitutions in the ZIKV/Macaca mulatta/UGA/MR-766_SM150-V8/1947, ZIKV/Homo sapiens/NGA/IbH-30656_SM21V1-V3/1968, and ZIKV/Homo Sapiens/PRI/PRVABC59/2015 stocks provided by BEI Resources. The sequenced amplicons confirmed the 12 nucleotide insertion/deletion, as well as the consensus variants that were detected by NGS sequencing in these regions of the selected strains, which were reported in our comparison.

### Deep Sequencing for Minor Variants

**Figure 1:**
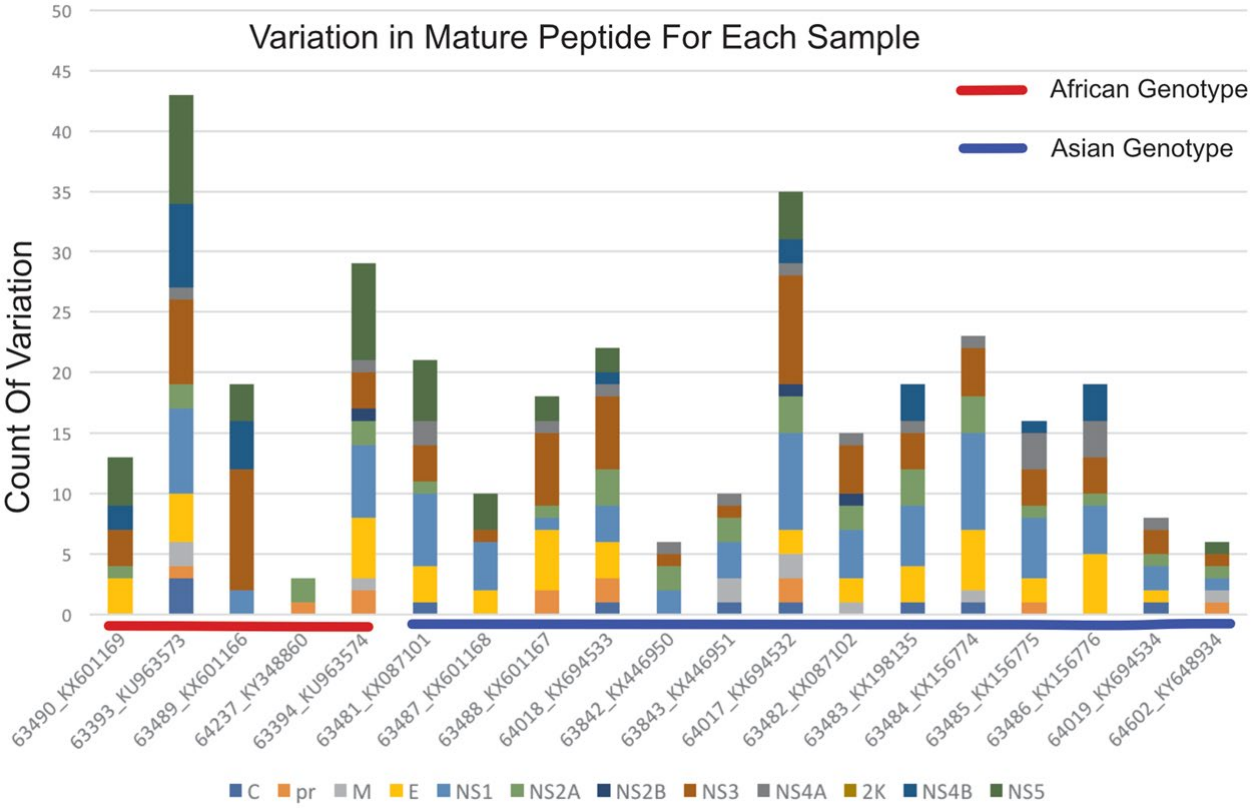
Minor variants detected in each coding ZIKV stock, by coding region. The number of statistically significant minor variants observed at a minimum of 20% of reads across the ZIKV genome. Values were normalized by the number of nucleotides in each gene.

Taking advantage of next-generation sequencing at a high read depth enables a better understanding of microevolution within an experimental system by identifying the minor variants (i.e. quasispecies) that exist within a population of viral genomes. We therefore set out to identify observed minor variants that displayed patterns which may contribute to viral adaptation to its host. To do so, we increased the frequency cutoff such that only minor variants in at least 20% of the mapped reads for any position were included in subsequent analyses. Given that ZIKV consists of a positive-sense single-stranded RNA genome, this cutoff should minimize the number of variants detected from viral transcripts as well as artifacts generated during PCR and/or reverse-transcription, and instead focus on those variants that are present at relatively high levels in the population of genomes within a given stock. To begin, we tabulated the number of minor variants compared to the consensus genome and calculated which coding regions contained the most minor variants after normalizing for gene length (Fig. 1). Interestingly, when both the African and Asian lineages were analyzed together, the cleaved pr region of the prM coding region contained the highest frequency of minor variants (0.053 variants per position). Separating the two lineages revealed that the prM coding region contained the highest frequency of minor variants in the Asian lineage (0.043 variants per position), while the E gene contained the highest frequency of minor variants in the African lineages (0.014 variants per position).

**Figure 2:**
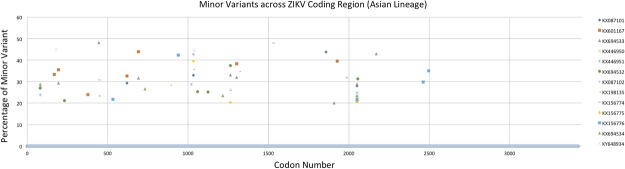
Positions of minor variants detected in Asian-lineage Zika viruses. The location of codons containing minor variants in strains belonging to the Asian lineage with the frequency threshold set at 20% (or more) of reads.

**Figure 3:**
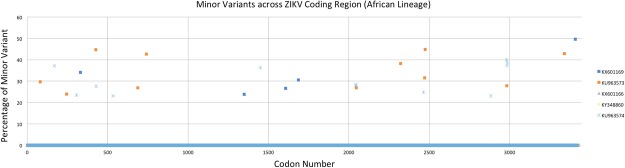
Positions of minor variants detected in African-lineage Zika viruses. The location of codons containing minor variants in strains belonging to the African lineage with the frequency threshold set at 20% (or more) of reads.

While examining the results across the coding region at the codon level, we found a larger overall number of total minor variants for each of the Asian strains (Fig. 2), than for strains belonging to the African lineages (Fig. 3). We also observed 11 minor variants shared among multiple strains at codon positions 81 (C), 168 (prM), 194 (prM), 451 (E), 533 (E), 620 (E), 691 (E), 1033 (NS1), 1263 (NS2A), 1303 (NS2A), and 2051 (NS3). Codons 81, 168, 451, 620, 691, 1033, 1263, and 1303 resulted in nonsynonymous amino acid substitutions while codons 194, 533, and 2051 caused no change in the amino acid sequence. All of the nonsynonymous changes, except for codon 168, were observed in at least two members of the Asian lineage. Interestingly, all but three of the nonsynonymous substitutions (620, 691, and 1263) were identical among all strains that contained minor variants at these positions (Table 5). The sample size is insufficient for providing statistical significance to these observations and the majority of the positions harboring variation appear to be mostly randomly distributed. Given their frequency and shared nature, it is unlikely that these positions have a negative effect on the viral population.

**Table 5:** Minor variants (>20%) observed in codons across Asian and African lineages and the effect on amino acid residue.

Strain Name	GenBank Accession	Lineage	Codon Position*										
			81	168	194	451	533	620	691	1033	1263	1303	2051
			C	prM	prM	E	E	E	E	NS1	NS2A	NS2A	NS3
Zika virus ZIKV/Macaca mulatta/UGA/MR-766/1947	KX601169	African											
Zika virus ZIKV/Macaca mulatta/UGA/MR-766_SM150-V8/1947	KU963573	African	I81M										
Zika virus ZIKV/Aedes africanus/SEN/DakAr41524/1984	KX601166	African											
Zika virus ZIKV/Aedes africanus/SEN/DAK-AR-41524_A1C1-V5/1984	KY348860	African											
Zika virus ZIKV/Homo sapiens/NGA/IbH-30656_SM21V1-V3/1968	KU963574	African		D168N			N/C						
Zika virus ZIKV/Homo Sapiens/PRI/PRVABC59/2015	KX087101	Asian						L620V		S1033N			N/C
Zika virus ZIKV/Aedes sp./MYS/P6-740/1966	KX601167	Asian		D168N	N/C			V620L	Y691H			A1303T	
Zika virus ZIKV/Aedes aegypti/MYS/P6-740/1966	KX694533	Asian	I81M		N/C				H691Y		V1263A	A1303T	N/C
Zika virus ZIKV/Aedes.sp/MEX/MEX_2-81/2016	KX446950	Asian											
Zika virus ZIKV/Aedes.sp/MEX/MEX_I-7/2016	KX446951	Asian	I81M										N/C
Zika virus ZIKV/Homo sapiens/THA/PLCal_ZV/2013	KX694532	Asian	I81M								A1263V		N/C
Zika virus ZIKV/Homo Sapiens/COL/FLR/2015	KX087102	Asian				D451E				S1033N			
Zika virus ZIKV/Homo sapiens/PAN/BEI-259634_V4/2016	KX198135	Asian								S1033N	A1263V		N/C
Zika virus ZIKV/Homo sapiens/PAN/CDC-259359_V1-V3/2015	KX156774	Asian				D451E				S1033N			N/C
Zika virus ZIKV/Homo sapiens/PAN/CDC-259249_V1-V3/2015	KX156775	Asian								S1033N	A1263V		N/C
Zika virus ZIKV/Homo sapiens/PAN/CDC-259364_V1-V2/2015	KX156776	Asian					N/C						N/C
Zika virus ZIKV/Homo sapiens/HND/R103451/2015	KX694534	Asian	I81M										N/C
Zika virus ZIKV/Aedes aegypti/MEX/MEX_I-44/2016	KY648934	Asian									V1263A		

^*^Positions are codon numbers in the polyprotein with genes indicated inside the parentheses.

N/C = No change in amino acid sequence.

### Recombination Analysis

RDP4 only detected a single possible recombination sequence across this dataset, KF383116.1/Unknown/ArD7117/SEN/1968 in the African lineage. This sequence showed statistically-significant evidence of being a potential recombinant with RDP, GENECONV, Maxchi, Chimaera, SiScan and 3Seq. In this case, KU955591.1/Aedes/41525-DAK/SEN/1984 was identified as the major parent and LC002520.1/Unknown/MR766-NIID/UGA/1947 was identified as the minor parent. The breakpoints were identified 322 bases apart at positions 5222–5544 with the parental and daughter strains all displaying high sequence similarity to each other.

### Phylogenetic Analysis

To better understand the evolutionary relationships between these sequences, we used the polyprotein coding region to reconstruct the phylogenetic relationships between the many ZIKV strains. Examination of the phylogenetic tree confirmed the existence of East African and West African lineages as was previously described^28^ (Supplementary Fig. S17). Among Asian lineage strains, the sequences responsible for the recent outbreak cluster very closely together. The basal relationships between the pre-outbreak FSM (2007 Micronesia), KHM/2010/FSS13025 (2010 Cambodia), PHL/2012/CPC 0740 (2012 Philippines), and THA/2014/SV0127 (2014 Thailand) strains to those associated with the recent outbreak also remains apparent in the phylogeny.

Using Bayesian methods, we calculated the ZIKV African and Asian lineages to have diverged from each other in the 17^th^ century, while the ancestor of all East and West African strains existed in the late 18^th^ century, and the most recent common ancestor for strains belonging to the Asian lineage existed in the late 19^th^ century. The East African sequences and West African sequences diverged in approximately 1816 and 1898 respectively, which differ only slightly from previously-reported dates^29^. Estimated mean evolutionary rates for the ZIKV genomes varied from 3.52E-04 (95% HPD 3.017E-4, 4.1099E-4) to 6.89E-04 (95% HPD 5.1318E-4, 8.6453E-4) nucleotide substitutions per site per year based on strict and relaxed molecular clock estimates with different priors. These dates are similar to those generated using linear regression (Supplementary Fig. S18).

Interestingly, a subset of the sequences had stronger than expected phylogenetic relationships due to either the time or place of strain isolation. For example, the Asian lineage sequences that were isolated during the 1966 Malaysian, 2010 Cambodian, or 2014 Thai outbreaks are all monophyletic and basal to the extant outbreak sequences. In addition, sequences from strains isolated from the Pacific islands are closer to each other than they are to those from other locations, including Latin America. In almost all cases, the sequences cluster together with the geographical origin of the virus. No clustering pattern was observed based on the source organism of the virus (i.e. the ZIKV sequence extracted from human did not cluster any differently from those extracted from mosquito).

### Selection Analysis

We used four algorithms to calculate global dN/dS values for the coding region of multiple ZIKV genomes belonging to either the Asian or African lineages (see Supplementary Table S14). Out of the 3424 codons that we included in our selection pressure analysis, 227, 579, and 233 were identified as undergoing negative (i.e. purifying) selection by one, two, or three algorithms, respectively. Conversely, there were 49, 2, and 1 codons with characteristics of positive (diversifying) selection identified by one, two, or three algorithms, respectively. Specifically, aligned codon 3163 was predicted by 3 algorithms as undergoing positive selection, while codons 2456 and 2808 were predicted by 2 algorithms as having a genetic signature of undergoing positive selection. After calculating dN/dS across the ZIKV coding region, we normalized the values according to the number of codons in each coding region (Supplementary Table S13). These results revealed that the 4% of M and 2.8% of NS1 codons in ZIKV had displayed evidence of undergoing positive selection, while 43% of codons in 2K and 35% of codons in E were identified as undergoing negative selection pressure. The strain names and GenBank accession numbers for sequences used in these phylogenetic, selection, and other comparative genomics analyses are provided (Supplementary Table S14).

## Discussion

Herein, we have identified consensus variants that differ between multiple sequence records for the same strain as well as minor variants within multiple virus stocks. The consensus sequences for stocks that are available through BEI Resources adequately represent the phylogenetic diversity of ZIKV, including the major ZIKV lineages (East African, West African, and Asian). The growing number of sequences from the recent outbreak are closely related while individual subclades share a high degree of phylogenetic relatedness. Recombination does not appear to play a significant role in the evolution of ZIKV, while evidence of selection pressure is present across viral proteins that are exposed to the host immune system.

It is important to note that the various Zika virus stocks have been sequenced at various points in time on various platforms. For example, the early isolates from Uganda, Malaysia, Micronesia, and possibly others were sequenced using Sanger technology^30,31^. Some of these same isolates have been sequenced again, together with viruses collected from the most recent outbreak using NGS instruments. These data together with other reported metadata, which describe the date and location of collection, are important to enable the accurate interpretation of computational results.

Our computational analyses show that these ZIKV strains are authentic and suitable for additional experiments. Although MR-766 (East African lineage) is the ZIKV reference sequence, other strains that belong to the Asian lineage would be much better suited as models for studying the recent Asian lineage-based outbreak. Moreover, it has been suggested previously that the 12-base deletion at positions 1435–1446 in the envelope of the MR-766 isolate seen in the stocks sequenced is because of extensive mouse brain passage of the isolate and therefore not a true representative of a natural strain^31^.

The fact that MR-766 is still one of the official Zika reference sequences is noteworthy because (1) MR-766 belongs to the African lineage (all recent outbreak strains fall into the Asian lineage) and (2) there are several bases in disagreement between the official reference sequence NC_012532.1 and other sequences derived from this isolate. We are confident in our data given the high-quality sequence read data and good coverage spanning the region of disagreement, which is reflected in the consensus coding region sequence. Moving forward, it will be important to take these sequence discrepancies into consideration since MR-766 is a common laboratory strain and has been used to establish ZIKV animal models^32^. The insertion/deletion reported here should be investigated further to determine whether its presence or absence affects viral binding and/or membrane fusion for viral entry in different hosts and cell types. Similar indels have been reported previously in other MR-766 sequences^27^. Most of the non-synonymous changes were located in the Envelope E and NS5 mature proteins. It is for these reasons that a transition to an Asian-lineage strain such as PRVABC59, especially for research in human-derived model systems should be carefully considered. Future phylogenetic and comparative genomics studies should ensure that any variants that are phylogenetically associated with the divergence between African and Asian sequences are not mistaken as “driver” mutations for increased neurovirulence.

We cannot definitively conclude that the predicted recombination event is accurate since both parent strains and the daughter strain are extremely similar to each other, and there is a lack of variation in this region among the three sequences. In addition, such a recombination event would require two template switch events in close proximity to each other to generate the predicted recombinant daughter sequence, which decreases the likelihood that this prediction is accurate.

The basal positioning and monophyletic nature of the Asian 1966 and 2007–2010 ZIKV sequences, suggest that these are unique ZIKV lineages that are not necessarily direct ancestors of the recent outbreak sequences. Instances where Brazilian sequences are the closest phylogenetic relative to strains isolated from recently-returned travelers to Italy likely indicate trans-Atlantic carriage of virus by a person that was later diagnosed clinically. In addition, the lack of clustering between viruses obtained from either host or vector organisms reflects the known biology of this virus and its continuous cycling between primates and mosquitoes, with very rare primate-to-primate transmission.

The deep sequencing analysis revealed novel insight into the seemingly non-random nature of minor variants that are present at higher frequencies (>20%). The fact that a subset of these minor variants is observed across multiple strains is of interest. How these high- and low-frequency variants contribute to viral evolution, host specificity, and/or the host innate and adaptive immune response over time is largely unknown and warrants additional investigation. However, one study reported an increase in cytopathic effects associated with the A1263V substitution among other mutations in a Brazilian strain collected during the recent ZIKV outbreak^33^. A resolved three-dimensional protein crystal structure of the ZIKV capsid (C) protein shows that the I81 residue, for which we identified a minor variant, contributes to hydrophobic interactions within the protein^34^. Although only one minor variant was reported for a given nucleotide position in each stock because of the cutoffs that we imposed, it is important to recognize that multiple co-circulating alleles are likely present in each virus stock. Our 20% frequency threshold and 0.05 corrected p-value cutoff removed many of the minor variants that were likely laboratory artifacts generated by random mutation, reverse transcription, amplification, and sequencing. The reported variants represent a sample of the detectable population of nucleotide variations present in the sequenced sample, which may consist of variants generated by natural virus variation and/or PCR artifacts. Additional work will be required to examine whether the minor variants present in these virus stocks are found at similar levels in a permissive animal host.

Positive selection pressure is primarily driven by the host immune response to viral proteins during infection. The relatively small number of codons undergoing positive selection in ZIKV genomes is somewhat surprising given that at least a subset of codons in other mosquito-borne Flavivirus genomes have been shown to be undergoing such pressure^35,36^. In addition, positive selection in RNA viruses transmitted through an insect vector is somewhat limited due a transmission cycle that involves alternating between different host species, together with the lack of an adaptive immune system in mosquitoes^37,38^. The high degree of negative selection in the M gene is an interesting result since the gene codes only for a structural protein that has minimal exposure to the host adaptive immune system. It is likely that the structural (and other) functions of the M protein are highly specialized and therefore sensitive to amino acid substitutions. The majority of genes that code for enzymes (e.g. NS3, NS5) were found to have an intermediate number of codons undergoing negative selection. We would expect that the amino acids in and around the active site(s) for these enzymes would have high negative selection. However, the majority of amino acid substitutions in other regions of these enzymatic proteins would likely have minimal effect on the function of these proteins.

The findings of this study will minimize the need for the scientific research community to separately authenticate the virus reagents being made available through public repositories such as BEI Resources. These data can be useful in designing and performing future comparative studies that focus on gaining a better understanding of the pathogenesis, neurotropism, spread, potential treatment, and prevention of ZIKV through comparative experiments.

## Electronic supplementary material

Supplemental Information

## Data Availability

All sequences generated as part of this study were submitted to GenBank under the Bioproject PRJNA314889.
