# Supplementary material for: Whole genome sequencing, variant analysis, phylogenetics, and deep sequencing of Zika virus strains
Source: Sci Rep. 2018 Oct 26;8:15843. doi: 10.1038/s41598-018-34147-7 (PMC6203802; doi:10.1038/s41598-018-34147-7)
Supplement: Supplementary file 1 — Supplemental Information [file 41598_2018_34147_MOESM1_ESM.docx]

**Title:**

Whole genome sequencing, variant analysis, phylogenetics, and deep sequencing of Zika virus strains

**Authors:**

Susmita Shrivastava^1^, Vinita Puri^1^, Kari A. Dilley^1+^, Erica Ngouajio^1++^, Jessica Shifflett^2^, Lauren Oldfield^1^, Nadia Fedorova^1^, Lihui Hu^1^, Torrey Williams^3^, Alan Durbin^3^, Paolo Amedeo^1^, Sujatha Rashid^2^, Reed S. Shabman^1+++^, Brett E. Pickett*^1^

**Supplementary Information:**

**African lineage analysis:**

The IbH-30656 isolate (NR-50066) of Zika virus was taken from the blood of a human in Ibadan, Nigeria, on September 9, 1968. We determined the consensus sequence for this stock (KU963574.2) and compared the coding region to the existing IbH-30656 sequence from NCBI (HQ234500). We found only one difference in the coding region as shown in Supplementary Table 2. This nucleotide change resulted in a synonymous substitution, hence no variation at protein level was detected. The JCVI sequence has an additional 88 bases at the 5` end and 427 bases at the 3` end compared to HQ234500.1

The Zika SEN/DAK-AR-41524/1984 (NR-50338) was isolated from a mosquito (*Aedes africanus*) in Kédougou, Senegal, on November 17, 1984. We sequenced two different stocks of this isolate as part of this study: KX198134.2 and KY348860.1. We compared one of these new sequences with others available from NCBI (KU955591.1, KU955592.1, KU955595.1, and KX601166.1) and found differences at 14 positions across the nucleotide sequences as shown in Supplementary Table 3. These 14 nucleotide changes resulted in a total of 3 amino acid differences across the entire polyprotein as shown in Supplementary Table 4. Two of the sequences, including the new one from JCVI are shorter at the 3` end by 7 bases, and also shorter at the 5` end compared to the sequences already available from NCBI.

**Asian lineage analysis:**

The Zika virus MYS/P6-740/1966 strain (NR-50245) was isolated from *Aedes aegypti* mosquitoes collected in Malaysia in July 1966. We sequenced one stock of this isolate within this study (KX694533.2) and compared it to earlier sequences deposited in NCBI (KX377336.1 and KX601167.1). Only 2 differences were observed at the nucleotide level between KX377336.1 and the stock provided by BEI Resources: KX694533.2, while 5 differences were detected between KX694533.2 and KX601167.1 as shown in Supplementary Table 5. For the polyprotein, five differences were observed among the three stocks, one each in the Capsid C, NS2A and NS5 protein and two differences in the Envelope E protein as shown in Table 6. The sequence of the 5` and 3` UTRs for the strain generated at JCVI as part of this study is incomplete.

The Zika virus COL/FLR/2015 (NR-50183) was isolated from the blood of a human in Barranquilla, Colombia in December 2015. After sequencing the NR-50283 stock (KX087102.2), we compared it to the GenBank sequence (KU820897.5) and found just one nucleotide difference as shown in Supplementary Table 7, resulting in a synonymous change, hence there was no variation the protein sequence.

The Zika virus MEX/MEX_I-44/2016 isolate (NR-50279) was identified in a sample of *Aedes aegypti* mosquitoes collected in Chiapas, Mexico in January 2016. We sequenced NR-50279 (KY648934.1) and compared it to the sequence that was already in NCBI (KX856011.1). Two differences at the nucleotide level were observed as shown in Supplementary Table 8, resulting in separate non-synonymous changes in the Envelope E protein as shown in Table 9.

The MEX/MEX_I-7/2016 (NR-50281) ZIKV isolate was identified from *Aedes aegypti* mosquitoes collected in Chiapas, Mexico by Ildefonso Fernandez-Salas of the Instituto Nacional De Salud Pública in January 2016. We sequenced NR-50281 virus stock (KX446951.2) and compared it to the sequence in NCBI (KX247632.1). This revealed three differences at the nucleotide level as shown in Supplementary Table 10. These three differences resulted in one amino acid substitution in the Envelope E protein as shown in Table 11.

**
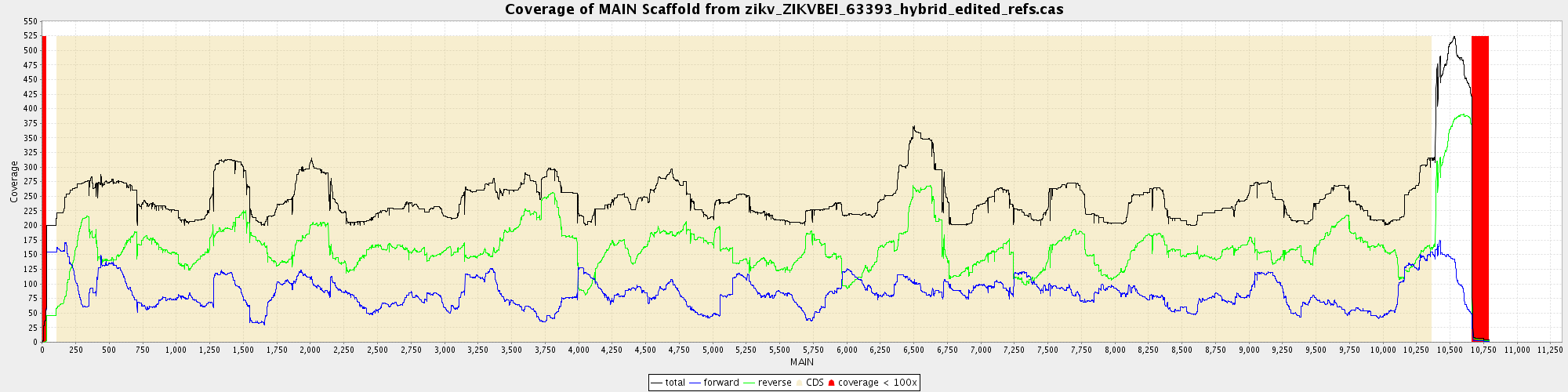
**

**Supplementary Figure S1:** A coverage plot of sequence reads generated from the ZIKV/Macaca mulatta/UGA/MR-766_SM150-V8/1947 virus stock. Forward, reverse, and total reads are represented by blue, green, and black lines respectively. The coding sequence (CDS) region is shaded with a yellow background while regions having coverage less than 100x are shaded with a red background.


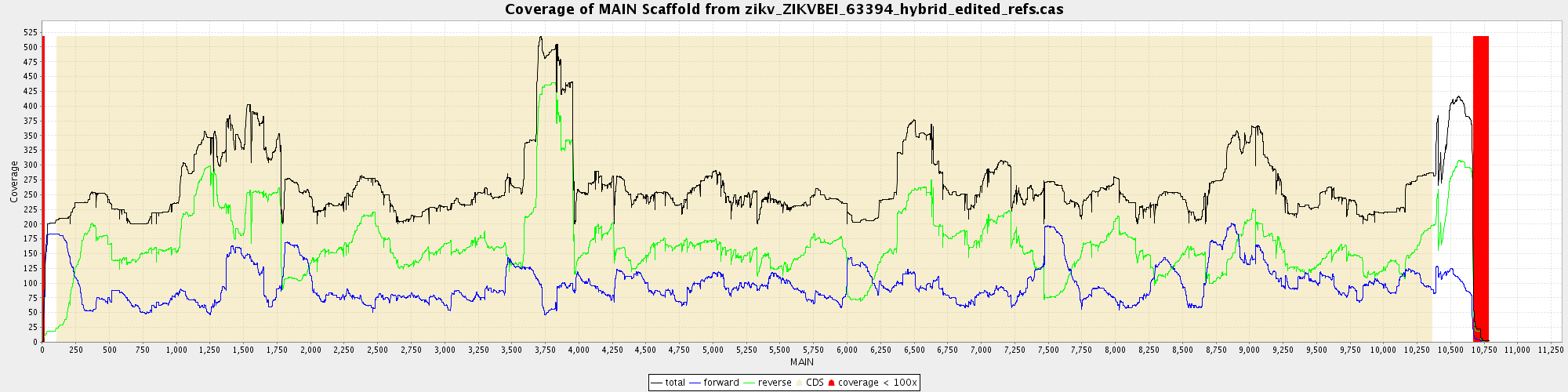


**Supplementary Figure S2:** A coverage plot of sequence reads generated from the ZIKV/Homo sapiens/NGA/IbH-30656_SM21V1-V3/1968 virus stock. Forward, reverse, and total reads are represented by blue, green, and black lines respectively. The coding sequence (CDS) region is shaded with a yellow background while regions having coverage less than 100x are shaded with a red background.


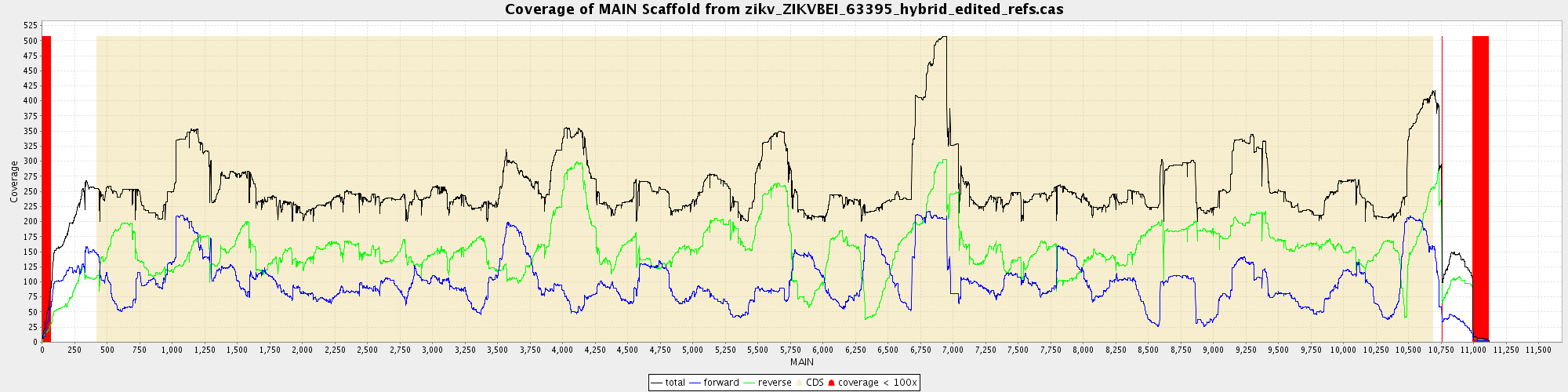
**Supplementary Figure S3:** A coverage plot of sequence reads generated from the ZIKV/Aedes africanus/SEN/DAK-AR-41524_A1C1-V2/1984 virus stock. Forward, reverse, and total reads are represented by blue, green, and black lines respectively. The coding sequence (CDS) region is shaded with a yellow background while regions having coverage less than 100x are shaded with a red background.

**
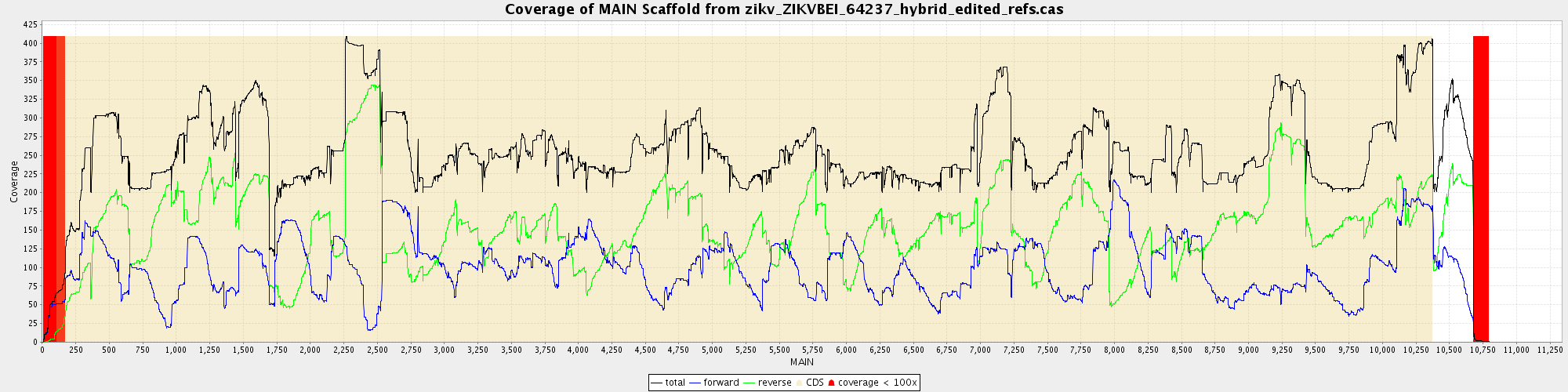
**

**Supplementary Figure S4:** A coverage plot of sequence reads generated from the ZIKV/Aedes africanus/SEN/DAK-AR-41524_A1C1-V5/1984 virus stock. Forward, reverse, and total reads are represented by blue, green, and black lines respectively. The coding sequence (CDS) region is shaded with a yellow background while regions having coverage less than 100x are shaded with a red background.


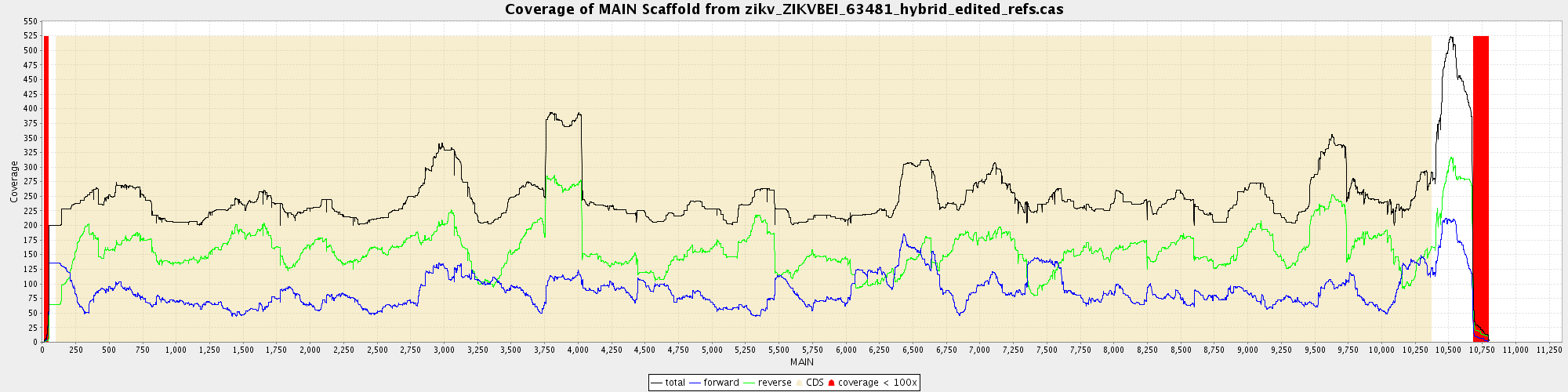


**Supplementary Figure S5:** A coverage plot of sequence reads generated from the ZIKV/Homo Sapiens/PR/PRVABC59/2015 virus stock. Forward, reverse, and total reads are represented by blue, green, and black lines respectively. The coding sequence (CDS) region is shaded with a yellow background while regions having coverage less than 100x are shaded with a red background.

**
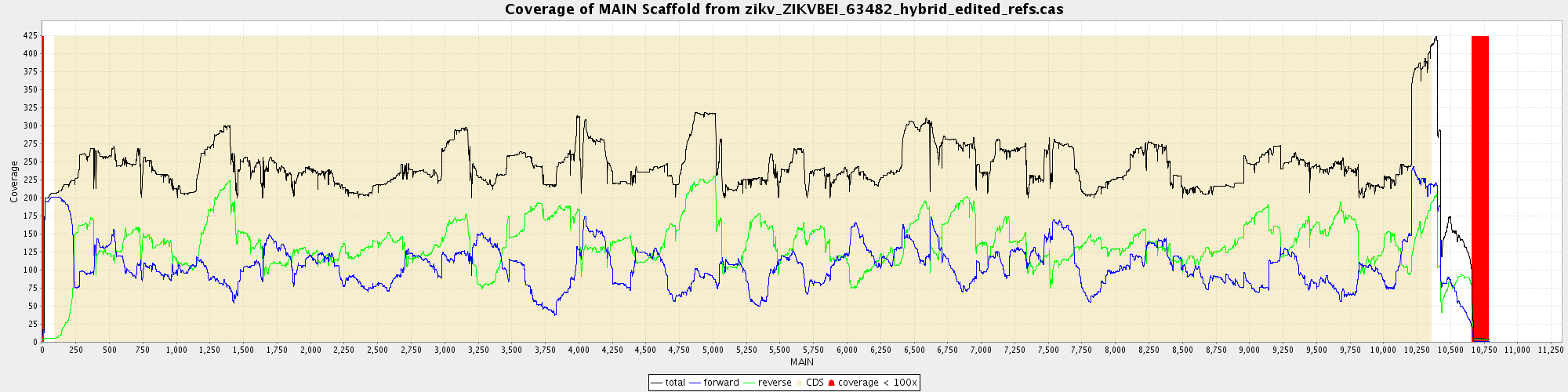
**

**Supplementary Figure S6:** A coverage plot of sequence reads generated from the ZIKV/Homo Sapiens/COL/FLR/2015 virus stock. Forward, reverse, and total reads are represented by blue, green, and black lines respectively. The coding sequence (CDS) region is shaded with a yellow background while regions having coverage less than 100x are shaded with a red background.


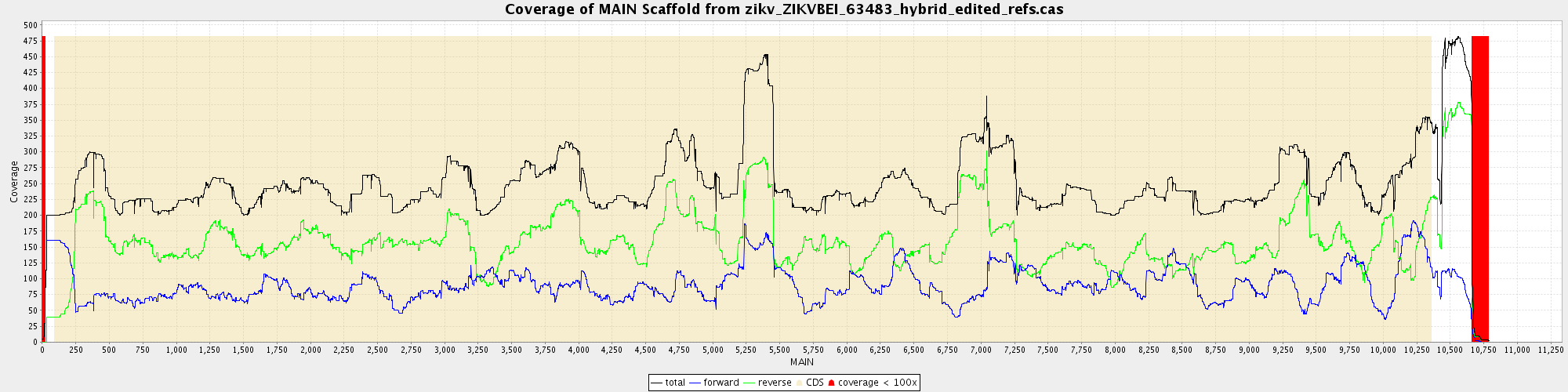


**Supplementary Figure S7:** A coverage plot of sequence reads generated from the ZIKV/Homo sapiens/PAN/BEI-259634_V1-Vx/2016 virus stock. Forward, reverse, and total reads are represented by blue, green, and black lines respectively. The coding sequence (CDS) region is shaded with a yellow background while regions having coverage less than 100x are shaded with a red background.


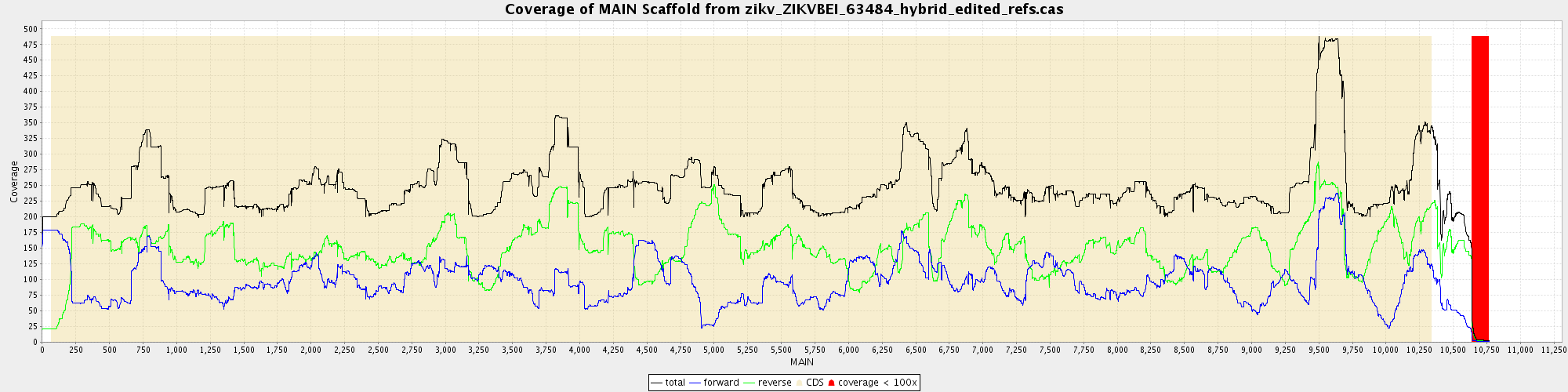


**Supplementary Figure S8:** A coverage plot of sequence reads generated from the ZIKV/Homo sapiens/PAN/CDC-259249_V1-Vx/2015 virus stock. Forward, reverse, and total reads are represented by blue, green, and black lines respectively. The coding sequence (CDS) region is shaded with a yellow background while regions having coverage less than 100x are shaded with a red background.


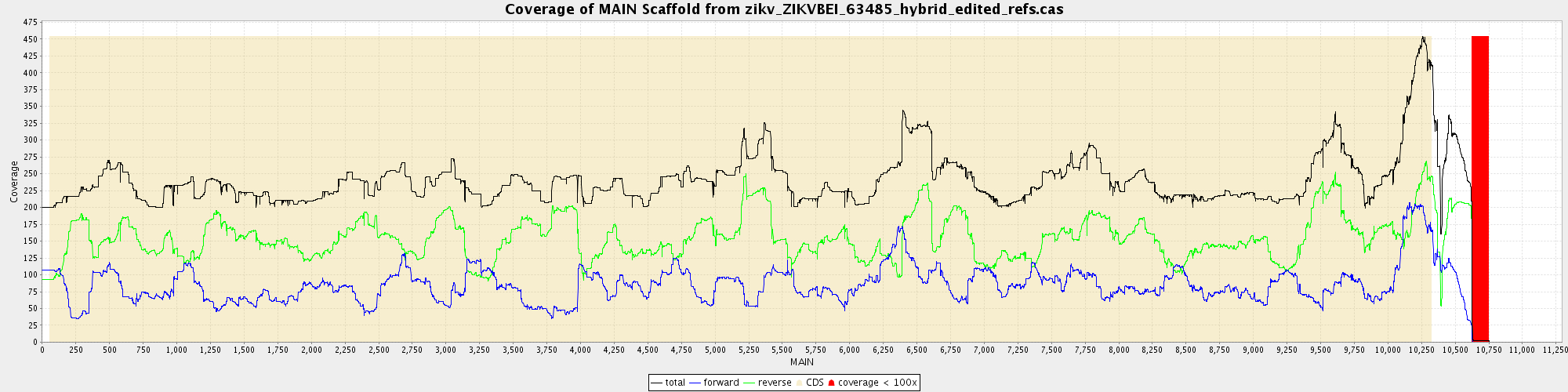


**Supplementary Figure S9:** A coverage plot of sequence reads generated from the Zika virus ZIKV/Homo sapiens/PAN/CDC-259249_V1-V3/2015 virus stock. Forward, reverse, and total reads are represented by blue, green, and black lines respectively. The coding sequence (CDS) region is shaded with a yellow background while regions having coverage less than 100x are shaded with a red background.

**
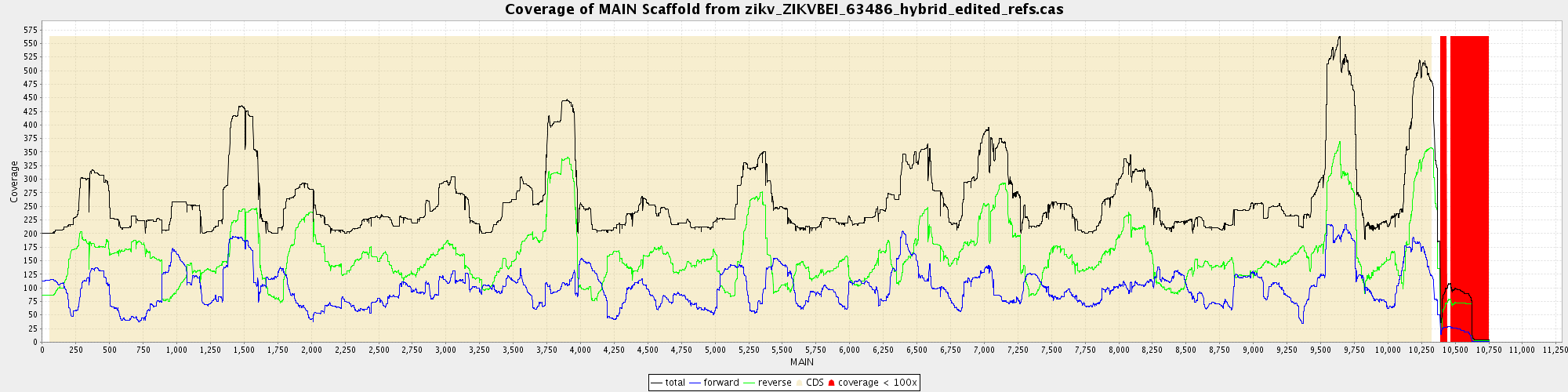
**

**Supplementary Figure S10:** A coverage plot of sequence reads generated from the ZIKV/Homo sapiens/PAN/CDC-259364_V1-Vx/2015 virus stock. Forward, reverse, and total reads are represented by blue, green, and black lines respectively. The coding sequence (CDS) region is shaded with a yellow background while regions having coverage less than 100x are shaded with a red background.


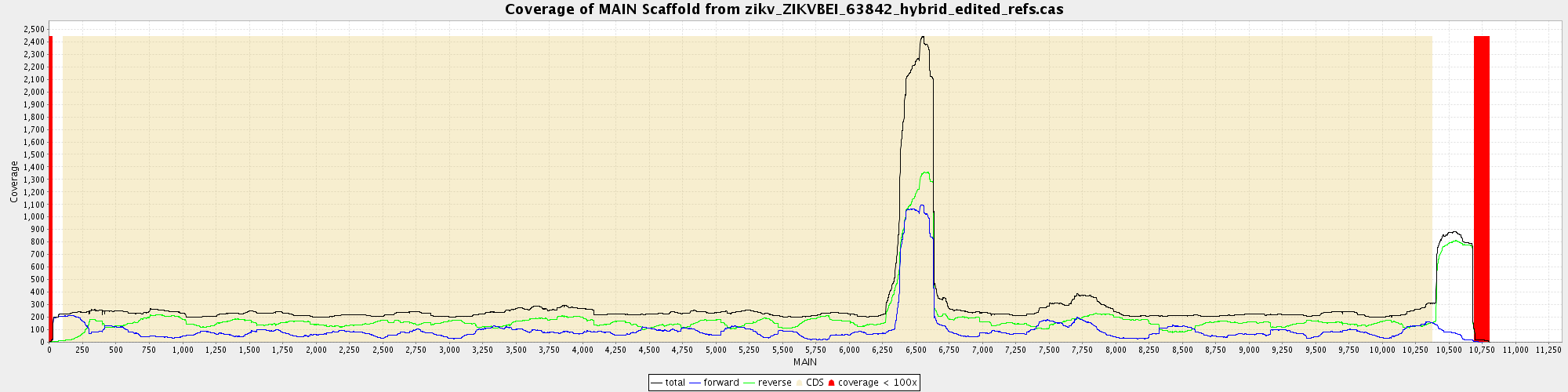


**Supplementary Figure S11:** A coverage plot of sequence reads generated from the ZIKV/Aedes.sp/MEX/MEX_2-81/2016 virus stock. Forward, reverse, and total reads are represented by blue, green, and black lines respectively. The coding sequence (CDS) region is shaded with a yellow background while regions having coverage less than 100x are shaded with a red background.


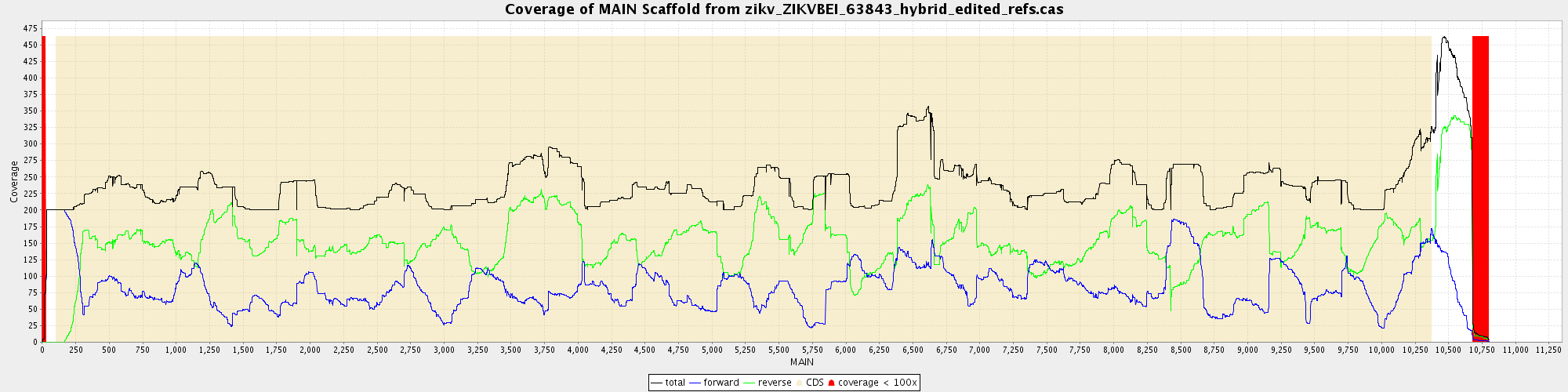


**Supplementary Figure S12:** A coverage plot of sequence reads generated from the ZIKV/Aedes.sp/MEX/MEX_I-7/2016 virus stock. Forward, reverse, and total reads are represented by blue, green, and black lines respectively. The coding sequence (CDS) region is shaded with a yellow background while regions having coverage less than 100x are shaded with a red background.


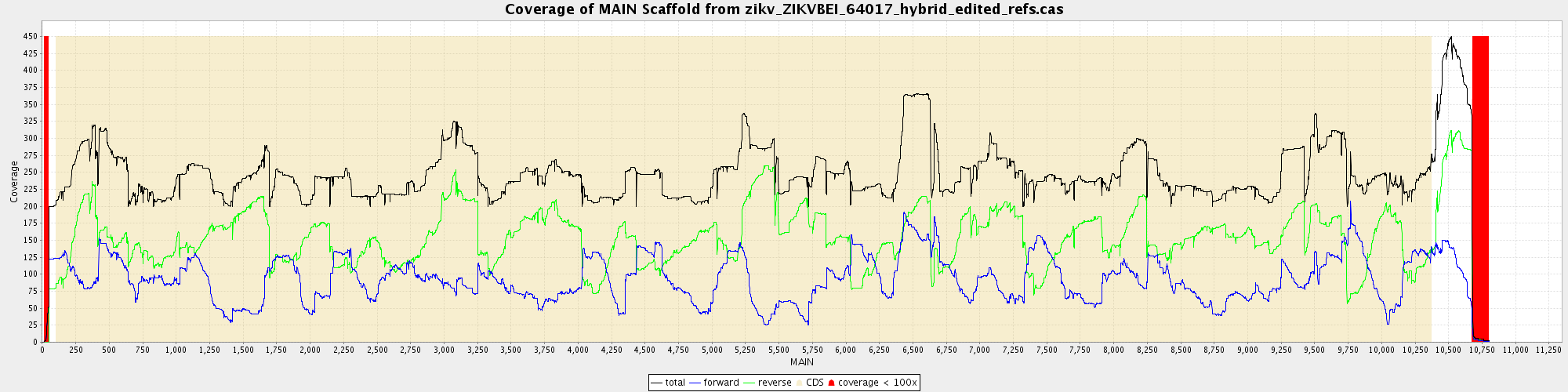


**Supplementary Figure S13:** A coverage plot of sequence reads generated from the ZIKV/Homo sapiens/THA/PLCal_ZV/2013 virus stock. Forward, reverse, and total reads are represented by blue, green, and black lines respectively. The coding sequence (CDS) region is shaded with a yellow background while regions having coverage less than 100x are shaded with a red background.

**
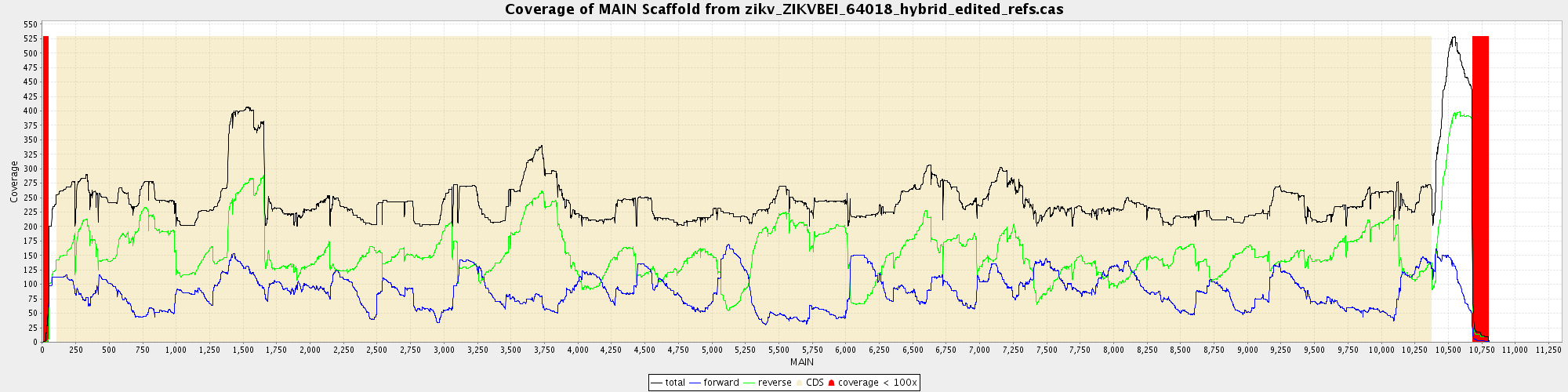
**

**Supplementary Figure S14:** A coverage plot of sequence reads generated from the ZIKV/Aedes aegypti/MYS/P6-740/1966 virus stock. Forward, reverse, and total reads are represented by blue, green, and black lines respectively. The coding sequence (CDS) region is shaded with a yellow background while regions having coverage less than 100x are shaded with a red background.


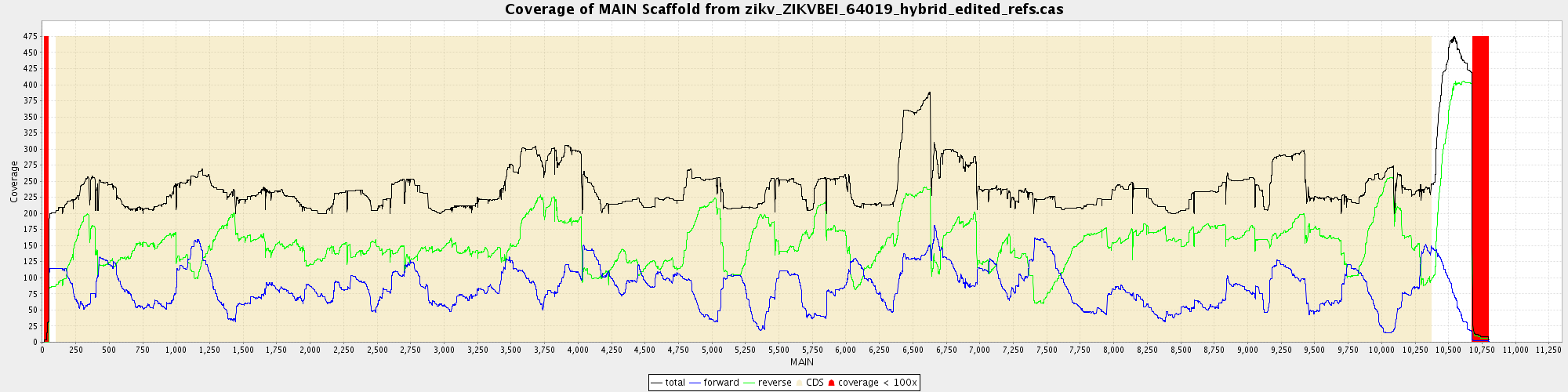


**Supplementary Figure S15:** A coverage plot of sequence reads generated from the Zika virus ZIKV/Homo sapiens/HND/R103451/2015 virus stock. Forward, reverse, and total reads are represented by blue, green, and black lines respectively. The coding sequence (CDS) region is shaded with a yellow background while regions having coverage less than 100x are shaded with a red background.


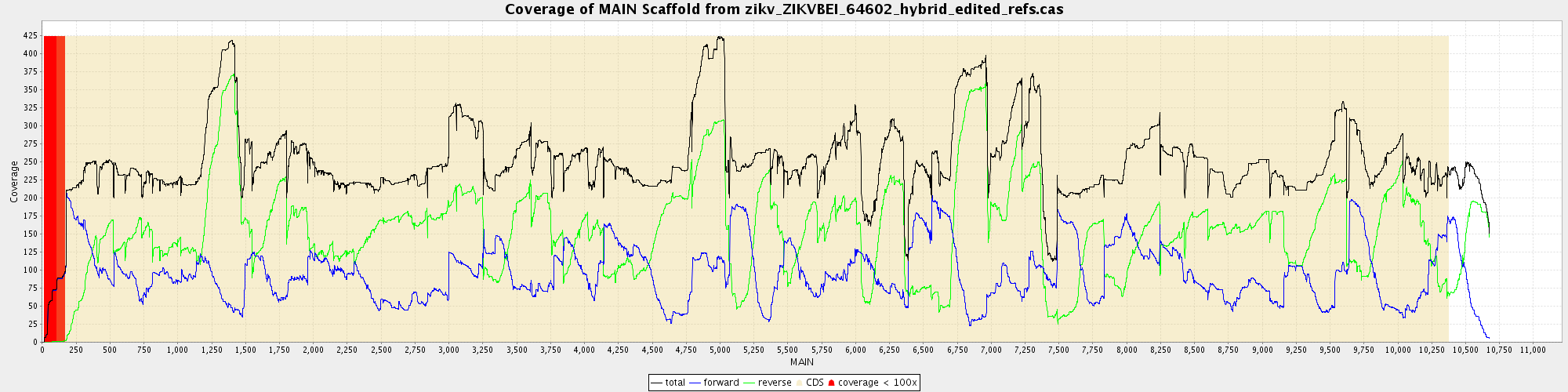


**Supplementary Figure S16:** A coverage plot of sequence reads generated from the Zika virus ZIKV/Aedes aegypti/MEX/MEX_I-44/2016 virus stock. Forward, reverse, and total reads are represented by blue, green, and black lines respectively. The coding sequence (CDS) region is shaded with a yellow background while regions having coverage less than 100x are shaded with a red background.

**
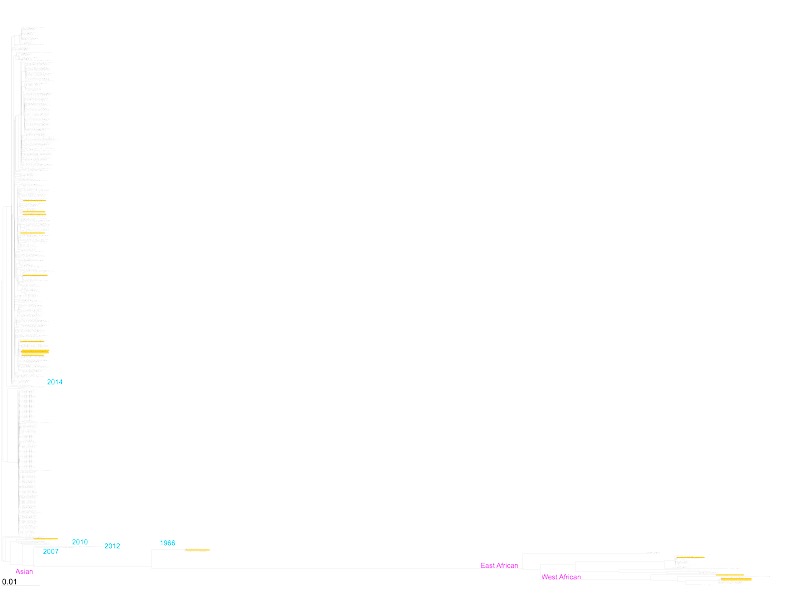
**

**Supplementary Figure S17**: Best-scoring Maximum Likelihood phylogenetic tree with bootstrap support values showing the evolutionary relationships between 448 Asian and African ZIKV sequences. The tree was rooted by year such that earlier isolates are located further from the root. The sequences highlighted in yellow were provided by BEI Resources for sequencing at JCVI. Magenta text indicates West African, East African, and Asian strains while blue text indicates the years of various outbreaks. Scale bar represents the number of nucleotide substitutions per site.

**
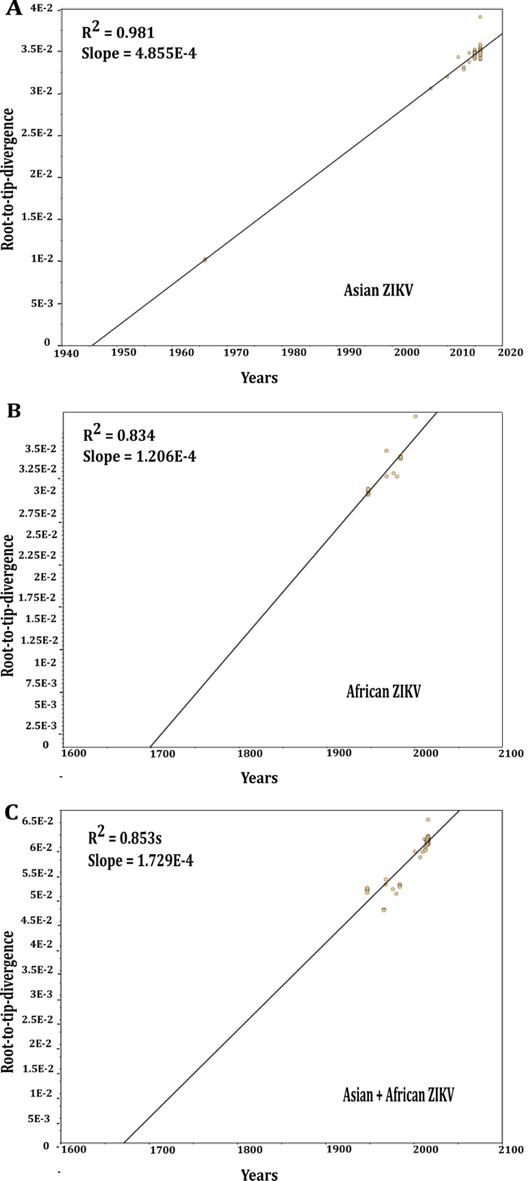
**

**Supplementary Figure S18:** Regression lines of the sampling date for each sequence against its genetic distance from the root-to-tip of the Maximum Likelihood tree of the Asian (top panel), African (middle panel), and combined (bottom panel) lineages generated using Path-O-gen.

**Supplementary Table S1:** List of all viruses with whole coding regions determined in this study.

| **Strain Name Revised** | **Accession Number** | **BEI Catalog** | **SISPA Sequencing Method** | **3' RACE** |
| --- | --- | --- | --- | --- |
| ZIKV/Macaca mulatta/UGA/MR-766_SM150-V8/1947 | KU963573 | NR-50065 | RNA and DNA | yes |
| ZIKV/Homo sapiens/NGA/IbH-30656_SM21V1-V3/1968 | KU963574 | NR-50066 | RNA and DNA | yes |
| ZIKV/Aedes africanus/SEN/DAK-AR-41524_A1C1-V5/1984 | KX198134 | NR-50338 | RNA and DNA | no |
| ZIKV/Homo sapiens/PRI/PRVABC59/2015 | KX087101 | NR-50240 | RNA | yes |
| ZIKV/Homo sapiens/COL/FLR/2015 | KX087102 | NR-50183 | RNA | yes |
| ZIKV/Homo sapiens/PAN/BEI-259634_V4/2016 | KX198135 | NR-50210 | RNA and DNA | yes |
| ZIKV/Homo sapiens/PAN/CDC-259359_V1-V3/2015 | KX156774 | NR-50219 | RNA and DNA | yes |
| ZIKV/Homo sapiens/PAN/CDC-259249_V1-V3/2015 | KX156775 | NR-50220 | RNA and DNA | yes |
| ZIKV/Homo sapiens/PAN/CDC-259364_V1-V2/2015 | KX156776 | NR-50221 | RNA and DNA | yes |
| ZIKV/Aedes.sp/MEX/MEX_2-81/2016 | KX446950 | NR-50280 | RNA and DNA | yes |
| ZIKV/Aedes.sp/MEX/MEX_I-7/2016 | KX446951 | NR-50281 | RNA and DNA | yes |
| ZIKV/Homo sapiens/THA/PLCal_ZV/2013 | KX694532 | NR-50234 | RNA | yes |
| ZIKV/Aedes aegypti/MYS/P6-740/1966 | KX694533 | NR-50245 | RNA | yes |
| ZIKV/Homo sapiens/HND/R103451/2015 | KX694534 | NR-50355 | RNA | yes |
| ZIKV/Aedes africanus/SEN/DAK-AR-41524_A1C1-V5/1984 | KY348860 | NR-50338 | RNA | no |
| ZIKV/Aedes aegypti/MEX/MEX_I-44/2016 | KY648934 | NR-50279 | RNA | no |

**Supplementary Table S2:** Comparison of different sequences of **NGA/Ibh-30656/1968** (NR-50066) with numbers representing the genome position in each sequence.

| **Alignment position** | **HQ234500.1** | **JCVI_KU963574.2_NR-50066 *** |
| --- | --- | --- |
| 4345 | T at 4257 | C at 4345 |
|  |  | Extra 88bp at 5` end and 427bp at 3` end compared to HQ234500.1 |

* sequenced at JCVI as part of the current work.

**Supplementary Table S3:** Comparison of different sequences of **SEN/DAK-AR-41524/1984** (NR-50338) with numbers representing the genome position in each sequence.

| **Alignment position** | **KU955591.1** | **KU955592.1** | **KU955595.1** | **JCVI_KY348860.1_NR-50338 *** | **KX601166.1** |
| --- | --- | --- | --- | --- | --- |
| 862 | T at 862 | C at 862 | C at 862 | C at 836 | C at 834 |
| 1586 | C at 1586 | C at 1586 | T at 1586 | C at 1560 | C at 1558 |
| 3816 | T at 3816 | T at 3816 | T at 3816 | C at 3790 | T at 3788 |
| 5299 | C at 5299 | T at 5299 | T at 5299 | C at 5273 | C at 5271 |
| 5990 | G at 5990 | A at 5990 | A at 5990 | G at 5964 | G at 5962 |
| 8191 | A at 8191 | A at 8191 | A at 8191 | G at 8165 | G at 8163 |
| 8347 | T at 8347 | T at 8347 | C at 8347 | T at 8321 | T at 8319 |
| 8810 | C at 8810 | T at 8810 | T at 8810 | C at 8784 | C at 8782 |
| 8839 | A at 8839 | G at 8839 | G at 8839 | A at 8813 | A at 8811 |
| 9076 | A at 9076 | A at 9076 | A at 9076 | G at 9050 | G at 9048 |
| 9799 | T at 9799 | T at 9799 | T at 9799 | C at 9773 | C at 9771 |
| 10651 | G at 10651 | A at 10651 | A at 10651 | A at 10625 | A at 10623 |
|  |  |  |  | Missing 26bp at 5` and 123bp at 3`end compared to KU955591.1 | Missing 7bp at 5` and 7bp at 3` end compared to KU955591.1 |

* sequenced at JCVI as part of the current work.

**Supplementary Table S4:** Comparison of different sequences of **SEN/DAK-AR-41524/1984** (NR-50338) with numbers representing the amino acid positions in the polyprotein for each sequence.

| **Alignment Position** | **KU955591.1** | **KU955592.1** | **KU955595.1** | **JCVI_KY348860_NR-50338 *** | **KX601166.1_NR-50338** |
| --- | --- | --- | --- | --- | --- |
| 1237 | V at 1237 | V at 1237 | V at 1237 | A at 1237 | V at 1237 |
| 1962 | V at 1962 | I at 1962 | I at 1962 | V at 1962 | V at 1962 |
| 2508 | Y at 2508 | Y at 2508 | H at 2508 | Y at 2508 | Y at 2508 |

* sequenced at JCVI as part of the current work.

**Supplementary Table S5:** Comparison of different sequences of **MYS/P6-740/1966** (NR-50245) with numbers representing the genome position in each sequence.

| **Alignment position** | **KX377336.1** | **JCVI_KX694533.2_NR-50245 *** | **KX601167.1** |
| --- | --- | --- | --- |
| 56 | G at 56 | A at 28 | G at 47 |
| 534 | A at 534 | A at 506 | G at 525 |
| 1444 | T at 1444 | T at 1416 | C at 1435 |
| 2178 | C at 2178 | C at 2150 | T at 2169 |
| 3895 | T at 3895 | T at 3867 | C at 3886 |
| 9804 | T at 9804 | C at 9776 | C at 9795 |

* sequenced at JCVI as part of the current work.

**Supplementary Table S6:** Comparison of different sequences of **MYS/P6-740/1966** (NR-50245) with numbers representing the amino acid positions in the polyprotein for each sequence.

| **Alignment position** | **KX377336.1** | **JCVI_KX694533.2_NR-50245 *** | **KX601167.1** |
| --- | --- | --- | --- |
| 143 | K at 143 | K at 143 | E at 143 |
| 446 | I at 446 | I at 446 | T at 446 |
| 691 | H at 691 | H at 691 | Y at 691 |
| 1263 | V at 1263 | V at 1263 | A at 1263 |
| 3233 | Y at 3233 | H at 3233 | H at 3233 |

* sequenced at JCVI as part of the current work.

**Supplementary Table S7:** Comparison of different sequences of **COL/FLR/2015** (NR-50183) with numbers representing the genome position in each sequence.

| **Alignment position** | **KU820897.5** | **JCVI_KX087102_NR-50183 *** |
| --- | --- | --- |
| 10736 | A at 10736 | G at 10719 |

* sequenced at JCVI as part of the current work.

**Supplementary Table S8:** Comparison of different sequences of **MEX/MEX_I-44/2016** (NR-50279) with numbers representing the genome position in each sequence.

| **Alignment position** | **KX856011** | **JCVI_KY648934.1_NR-50279 *** |
| --- | --- | --- |
| 3206 | A at 3206 | C at 3192 |
| 3878 | C at 3878 | T at 3864 |
|  |  | Missing 13bp from 5` end and 83bp from 3` end compared to KX856011.1 |

* sequenced at JCVI as part of the current work.

**Supplementary Table S9:** Comparison of different sequences of **MEX/MEX_I-44/2016** (NR-50279) with numbers representing the amino acid positions in the polyprotein for each sequence.

| **Alignment position** | **KX856011** | **JCVI_KY648934.1_NR-50279 *** |
| --- | --- | --- |
| 1039 | K at 1039 | T at 1039 |
| 1263 | A at 1263 | V at 1263 |

* sequenced at JCVI as part of the current work.

**Supplementary Table S10:** Comparison of different sequences of **MEX/MEX_I-7/2016** (NR-50281) with numbers representing the genome position in each sequence.

| **Alignment position** | **KX247632.1** | **JCVI_KX446951.1_NR-50281 *** |
| --- | --- | --- |
| 947 | C at 947 | T at 930 |
| 3895 | C at 3895 | T at 3878 |
| 10774 | C at 10774 | T at 10757 |
|  |  | Missing 17bp at 5` end and has an extra 30bp at 3` end compared to KX247632.1 |

* sequenced at JCVI as part of the current work.

**Supplementary Table S11:** Comparison of different sequences of **MEX/MEX_I-7/2016** (NR-50281) with numbers representing the amino acid positions in the polyprotein for each sequence.

| **Alignment position** | **KX247632.1** | **JCVI_KX446951.1_NR-50281 *** |
| --- | --- | --- |
| 1263 | A at 1263 | V at 1263 |

* sequenced at JCVI as part of the current work.

**Supplemental Table S12:** Summarized read coverage for each strain sequenced in this study

| **Strain name** | **GenBank accession number** | **BEI catalog** | **Published consensus coverage** | | | **# assembled reads in published assembly** | **Full data coverage** | | |
| --- | --- | --- | --- | --- | --- | --- | --- | --- | --- |
|  |  |  | **min** | **max** | **avg** |  | **min** | **max** | **avg** |
| ZIKV/Macaca mulatta/UGA/MR-766_SM150-V8/1947 | KU963573 | NR-50065 | 200 | 371 | 243.72 | 12685 | 7 | 134,851 | 15425.48 |
| ZIKV/Homo sapiens/NGA/IbH-30656_SM21V1-V3/1968 | KU963574 | NR-50066 | 200 | 518 | 263.69 | 13460 | 370 | 74743 | 10412.31 |
| ZIKV/Aedes africanus/SEN/DAK-AR-41524_A1C1-V5/1984 | KX198134 | NR-50338 | 200 | 507 | 259.04 | 13455 | 1 | 178567 | 11524.38 |
| ZIKV/Homo Sapiens/PRI/PRVABC59/2015 | KX087101 | NR-50240 | 200 | 393 | 244.52 | 13275 | 5 | 615270 | 41426.36 |
| ZIKV/Homo Sapiens/COL/FLR/2015 | KX087102 | NR-50183 | 200 | 413 | 245.33 | 13207 | 11 | 139131 | 7806.4 |
| ZIKV/Homo sapiens/PAN/BEI-259634_V4/2016 | KX198135 | NR-50210 | 200 | 453 | 248.24 | 14322 | 6 | 243703 | 25271.68 |
| ZIKV/Homo sapiens/PAN/CDC-259359_V1-V3/2015 | KX156774 | NR-50219 | 199 | 488 | 249.84 | 14148 | 6 | 259162 | 29461.8 |
| ZIKV/Homo sapiens/PAN/CDC-259249_V1-V3/2015 | KX156775 | NR-50220 | 199 | 454 | 239.82 | 13874 | 6 | 376253 | 25624.1 |
| ZIKV/Homo sapiens/PAN/CDC-259364_V1-V2/2015 | KX156776 | NR-50221 | 189 | 563 | 263.60 | 14469 | 7 | 222185 | 18715.9 |
| ZIKV/Aedes.sp/MEX/MEX_2-81/2016 | KX446950 | NR-50280 | 200 | 2443 | 286.08 | 13949 | 8 | 143341 | 22826.14 |
| ZIKV/Aedes.sp/MEX/MEX_I-7/2016 | KX446951 | NR-50281 | 200 | 357 | 234.16 | 11108 | 6 | 168206 | 19554.4 |
| ZIKV/Homo sapiens/THA/PLCal_ZV/2013 | KX694532 | NR-50234 | 200 | 366 | 246.26 | 12171 | 12 | 47255 | 4736.37 |
| ZIKV/Aedes aegypti/MYS/P6-740/1966 | KX694533 | NR-50245 | 200 | 407 | 242.23 | 12322 | 9 | 25249 | 4303.48 |
| ZIKV/Homo sapiens/HND/R103451/2015 | KX694534 | NR-50355 | 200 | 389 | 238.10 | 11982 | 25 | 63158 | 6681.59 |
| ZIKV/Aedes africanus/SEN/DAK-AR-41524_A1C1-V5/1984 | KY348860 | NR-50338 | 66 | 409 | 256.60 | 12393 | 4 | 39301 | 2205.42 |
| ZIKV/Aedes aegypti/MEX/MEX_I-44/2016 | KY648934 | NR-50279 | 75 | 424 | 253.65 | 13898 | 0 | 28129 | 1035.51 |

**Supplementary Table S13:** Number of codons calculated to be under positive selection, by mature peptide (normalized by mature peptide length).

| **Mat peptide** | **# of hits** | **Start** | **End** | **Normalized # hits per position** |
| --- | --- | --- | --- | --- |
| **C** | **1** | **1** | **112** | **0.009** |
| **pr** | **2** | **131** | **223** | **0.022** |
| **M** | **3** | **224** | **298** | **0.040** |
| **E** | **6** | **299** | **802** | **0.012** |
| **NS1** | **10** | **803** | **1154** | **0.028** |
| **NS2A** | **3** | **1155** | **1380** | **0.013** |
| **NS2B** | **1** | **1381** | **1510** | **0.008** |
| **NS3** | **9** | **1511** | **2127** | **0.015** |
| **NS4A** | **0** | **2128** | **2254** | **0.000** |
| **2K** | **0** | **2255** | **2277** | **0.000** |
| **NS4B** | **4** | **2278** | **2528** | **0.016** |
| **NS5** | **13** | **2529** | **3431** | **0.014** |

**Supplementary Table S14:** List of accession numbers and strain names that were used as input to the computational analyses for this study.

| **GenBank Accession Number** | **Strain Name** |
| --- | --- |
| KY765321.1 | Human/NIC/4886_12A1_SP/2016 |
| KY765318.1 | Human/NIC/4886_12A1/2016 |
| KY765317.1 | Human/NIC/7252_12A1/2016 |
| KY765322.1 | Human/NIC/7252_12A1_SP/2016 |
| KY765325.1 | Human/NIC/5005_13A1/2016 |
| KY765327.1 | Human/NIC/5005_13A1_SP/2016 |
| KY765323.1 | Human/NIC/6188_13A1/2016 |
| KY765326.1 | Human/NIC/6188_13A1_SP/2016 |
| KX694534.1 | Human/R103451/HND/2015 |
| KX262887.1 | Human/103451/HND/2016 |
| KU501216.1 | Human/103344/GTM/2015 |
| KU501217.1 | Human/8375/GTM/2015 |
| KY648934.1 | Aedes/MEX/MEX_I-44/2016 |
| KX856011.1 | Aedes/MEX_I-44/MEX/2016 |
| KX446951.1 | Aedes/MEX_I-7/MEX/2016 |
| KX247632.1 | Human/MEX_I_7/MEX/2015 |
| KX446950.1 | Aedes/MEX_2-81/MEX/2016 |
| KU870645.1 | Human/FB-GWUH_2016/USA/2016 |
| KX766029.1 | Human/R116265/MEX/2016 |
| KY765320.1 | Human/NIC/6406_13A1_SP/2016 |
| KY765324.1 | Human/NIC/8610_13A1/2016 |
| KX893855.1 | Human/UF-2/VEN/2016 |
| KX702400.1 | Human/UF-1/VEN/2016 |
| KX247646.1 | Human/UF-1/COL/2016 |
| KX087102.1 | Human/FLR/COL/2015 |
| KU820897.5 | Human/FLR/COL/2015 |
| KX198135.1 | Human/BEI-259634_V4/PAN/2016 |
| KX156776.1 | Human/CDC-259364_V1-V2/PAN/2015 |
| KX156774.1 | Human/CDC-259359_V1-V3/PAN/2015 |
| KX156775.1 | Human/CDC-259249_V1-V3/PAN/2015 |
| KU922923.1 | Human/MEX-InDRE-Lm/MEX/2016 |
| KU922960.1 | Human/InDRE-Sm-MEX/2016 |
| KU647676.1 | Human/MRS_OPY_Martinique_PaRi_2015/MTQ/2015 |
| KX811222.1 | Unknown/Brazil_2015_MG/BRA/2015 |
| KX280026.1 | Human/Paraiba_01/BRA/2015 |
| KX806557.1 | Human/TS17-2016/AUS/2016 |
| KX369547.1 | Human/PF13-251013-18/PYF/2013 |
| KJ776791.2 | Human/H-PF-2013/PYF/2013 |
| KX197192.1 | Human/PE243/BRA/2015 |
| KU321639.1 | Human/SPH2015/BRA/2015 |
| KU509998.3 | Human/Haiti-1225-2014/HTI/2014 |
| KX051563.1 | Human/Haiti-1-2016/USA/2016 |
| KU527068.1 | Human/Natal-RGN/BRA/2015 |
| KX197205.1 | Human/Isolate-9/BRA/2015 |
| KU991811.1 | Human/Brazil-INMI1/ITA/2016 |
| KU729218.1 | Human/BeH828305/BRA/2015 |
| KX601168.1 | Human/PRVABC59/PRI/2015 |
| KX087101.2 | Human/PRVABC59/PRI/2015 |
| KU501215.1 | Human/PRVABC59/PRI/2015 |
| KX377337.1 | Human/PRVABC-59/PRI/2015 |
| KU365778.1 | Human/BeH819015/BRA/2015 |
| KU758877.1 | Human/17271/GUF/2015 |
| KU707826.1 | Human/SSABR1/BRA/2015 |
| KU365779.1 | Human/BeH819966/BRA/2015 |
| KU365777.1 | Human/BeH818995/BRA/2015 |
| KU365780.1 | Human/BeH815744/BRA/2015 |
| KU312312.1 | Human/Z1106033/SUR/2015 |
| KU926309.1 | Human/Rio-U1/BRA/2016 |
| KX879603.1 | Human/SN062/ECU/2016 |
| KX879604.1 | Human/SN089/ECU/2016 |
| KU497555.1 | Human/ZKV2015/BRA/2015 |
| KU729217.2 | Human/BeH823339/BRA/2015 |
| KU926310.1 | Human/Rio-S1/BRA/2016 |
| KX766028.1 | Human/R114916/DOM/2016 |
| KU820898.1 | Human/GZ01/CHN/2016 |
| KU740184.2 | Human/GD01/CHN/2016 |
| KU761564.1 | Human/GDZ16001/CHN/2016 |
| KX266255.1 | Human/ZIKV_SMGC-1/CHN/2016 |
| KX253996.1 | Human/ZKC2/CHN/2016 |
| KU820899.2 | Human/ZJ03/CHN/2016 |
| KX117076.1 | Human/Zhejiang04/CHN/2016 |
| KX673530.1 | Human/PHE_semen_Guadeloupe/GBR/2016 |
| KU853012.1 | Human/PD1-DOM/ITA/2016 |
| KU853013.1 | Human/PD2-DOM/ITA/2016 |
| KX520666.1 | Human/HS-2015-BA-01/BRA/2015 |
| KX827309.1 | Human/ZKA-16-291/SGP/2016 |
| KX813683.1 | Human/ZKA-16-097/SGP/2016 |
| KU681081.3 | Human/SV0127-14/THA/2014 |
| KU744693.1 | Human/VE_Ganxian/CHN/2016 |
| KX694532.1 | Human/PLCal_ZV/THA/2013 |
| KU955593.1 | Human/FSS13025/KHM/2010 |
| EU545988.1 | Human/FSM/FSM/2007 |
| KU681082.3 | Human/CPC-0740/PHL/2012 |
| KX694533.1 | Aedes/P6-740/MYS/1966 |
| KX377336.1 | Aedes/P6-740/MYS/1966 |
| KX601167.1 | Aedes/P6-740/MYS/1966 |
| KX601166.2 | Aedes/DakAr41524/SEN/1984 |
| KY348860.1 | Aedes/SEN/DAK-AR-41524_A1C1-V5/1984 |
| KX198134.1 | Aedes/DAK-AR-41524_A1C1-V2/SEN/1984 |
| KU955591.1 | Aedes/41525-DAK/SEN/1984 |
| KU955592.1 | Aedes/41662-DAK/SEN/1984 |
| KU955595.1 | Aedes/41671-DAK/SEN/1984 |
| KF383116.1 | Unknown/ArD7117/SEN/1968 |
| KU963574.1 | Human/IbH-30656_SM21V1-V3/NGA/1968 |
| KX830960.1 | Monkey/ATCC:VR-84-MR766/UGA/1947 |
| KU720415.1 | Unknown/MR-766/UGA/1947 |
| KX377335.1 | Unknown/MR-766/UGA/1947 |
| LC002520.1 | Unknown/MR766-NIID/UGA/1947 |
| KX601169.1 | Monkey/MR-766/UGA/1947 |
| KU955594.1 | Monkey/MR-766/UGA/1947 |
| KU963573.1 | Monkey/MR-766_SM150-V8/UGA/1947 |
| NC_012532.1 | Monkey/UGA/MR-766/1947 |
| KF383118.1 | Unknown/ArD157995/SEN/2001 |
| KF268948.1 | Aedes/ARB13565/CAR/1976 |
| KF268949.1 | Aedes/ARB15076/CAR/1980 |
| DQ859059.1 | Unknown/MR_766/UGA/1947 |
